# Supplementary material for: CORK1, A LRR-Malectin Receptor Kinase, Is Required for Cellooligomer-Induced Responses in Arabidopsis thaliana
Source: Cells. 2022 Sep 22;11(19):2960. doi: 10.3390/cells11192960 (PMC9563578; doi:10.3390/cells11192960)
Supplement: Supplementary file 1 [file cells-11-02960-s001.zip › 20220908 Supplementary Figures+Supplementary Tables.pdf]

Supplementary Information

CORK1, a LRR-Malectin Receptor Kinase, is required for Cellooligomer-induced Responses in *Arabidopsis thaliana*

Yu-Heng Tseng[1], Sandra S. Scholz[1], Judith Fliegmann[2], Thomas Krüger[3], Akanksha Gandhi[1], Alexandra C. U. Furch[1], Olaf Kniemeyer[3,4], Axel A. Brakhage[3], Ralf Oelmüller[1*]

[1]Matthias Schleiden Institute of Genetics, Bioinformatics and Molecular Botany, Department of Plant Physiology, Friedrich-Schiller-University Jena, Jena, Germany
[2]Center for Plant Molecular Biology (ZMBP), University of Tübingen, Tübingen, Germany
[3]Department of Molecular and Applied Microbiology, Leibniz Institute for Natural Product Research and Infection Biology - Hans Knöll Institute (Leibniz-HKI), Jena, Germany
[4]Department of Microbiology and Molecular Biology, Institute of Microbiology, Friedrich Schiller University, Jena, Germany

*** Correspondence:**
Ralf Oelmüller
b7oera@uni-jena.de

```
                   10         20         30         40         50
           ....|....| ....|....| ....|....| ....|....| ....|....|
X.laevis   -MLSIRTVLG PLATILLTVL GPFGAHGSGL ADKVIWAVNA GGESHVDVHG
CORK1      FNFFVNCGGR DIRSS---SG ALYEKDEGAL GPATFFVSK- TQRWAVSNVG
AT1G56120  YNFSINCGGP EIRSV---SG ALFEKEDADL GPASFVVSA- AKRWAASSVG
AT1G56130  SDFSINCGGP EKRSV---TG ALFEREDEDF GPASFFVSA- GQRWAASSVG
AT1G56140  SDFSINCGGP EIRSV---TE AVFEREDEDL GPASFVVSA- GQRWAASSVG
AT1G07650  YKLYINCGGG EVKVDKEIT- --YQADDEPK G-ASMYVLGA NKRWALSSTG
AT1G29720  RFLHINCGGE EVSIRNSLGK ITYQTDNSRQ TNAASNQQ-- FDYWGVSNTG
AT1G29730  RSLHINCGGP DVTIENSRGR FLYEGDNYGL TGSATNYY-- RKNWGYSNTG
AT1G29740  RSLHINCGGP DVTIENSRGR FLYEGDNYGL TGSATNYY-- GKNWGFSNTG
AT1G29750  SCLHVNCGGS DMYVKEKKTK ELYEGDGNVE GGAAKYFLKP DANWGFSSTG
AT1G53420  NALHINCGGD EMSIN----G TIYESDKYDR LESWYESR-- -NGWFSNNVG
AT1G53430  SSLFINCGGS RLKIG----K DTYTDDLNSR GQSTFSSVS- -ERWGYSSSG
AT1G53440  SSLFINCGGN RLKVD----K DEYADDLNKR GASTFSSVS- -ERWGYSSSG
AT3G14840  YGLHINCGGN EITSN----E TKYDADTWDT PG-YYDSK-- -NGWVSSNTG
AT2G22610  TIMFINAGGD DSKVL----- ---------- ---------- DSELNISRDD
AT1G72250  PVISINSGSI STDVT----- ---------- ---------- VEDVTFLKDE

                   60         70         80         90        100
           ....|....| ....|....| ....|....| ....|....| ....|....|
X.laevis   IHYRKDPLEG RVGRASDYGM KLPILRSNPE DQVLYQTERY NEDSFGYDIP
CORK1      LFTGSNSNQY IALSATQ--- -----FANTS DSELFQSARL SASSLRYYGL
AT1G56120  NFAGSSNNIY IATSLAQ--- -----FINTM DSELFQSARL SASSLRYYGL
AT1G56130  LFAGSSNNIY IATSQSQ--- -----FVNTL DSELFQSARL SASSVRYYGL
AT1G56140  LFAGSSNNIY ISTSQSQ--- -----FVNTL DSELFQSARL SASSLRYYGL
AT1G07650  NFMDNDDDAD EYTVQNTS-- R-LSVNASSP SFGLYRTARV SPLSLTYYGI
AT1G29720  DFTDDNSDHD EYYTSTN--- ----LTLSGD YPDLYKTARR SALSLVYYAF
AT1G29730  DFMDDAITED TYTVSSE--- ----SAVSAK YPDLYQNARR SPLSLAYYAF
AT1G29740  DFMDDAITED TYTVSSE--- ----SAVSAK YPDLYQNARR SPLSLAYFAI
AT1G29750  DFMDDNN--- ---FQNTR-- F-TMFVPASN QSDLYKSARI APVSLTYFHA
AT1G53420  VFVDDKHVPE RVTIESNS-- SELNV----V DFGLYTQARI SAISLTYYAL
AT1G53430  VWLGKEDAGY LATDRFN--- -----LINGS TPEYYKTARL SPQSLKYYGL
AT1G53440  AWLGNDGATY LATDTFN--- -----LINES TPEYYKTARL ASQSLKYYGL
AT3G14840  NFLDDDRTNN GKSKWSNS-- SELKITNSSI DFRLYTQARL SAISLTYQAL
AT2G22610  YFEGGDVLRT EESIVEA--- --------GD FPFIYQSARV G--NFCYQLN
AT1G72250  FFSGGESI-T TDAVVGN--- --------ED EILLYQTARL G--NFAYKFQ

                  110        120        130        140        150
           ....|....| ....|....| ....|....| ....|....| ....|....|
X.laevis   IKEEGEYVLV LKFAEVYFAQ SQQ------- -KVFDVRVNG HTVVKDLDIF
CORK1      GLENGGYSVT VQFAEIQIQG SNT--WKSLG RRIFDIYVQG KLVEKDFDMQ
AT1G56120  GLENGGYTVT LQFAEVQIEG SNS--WKGIG RRRFNIYVQG RLVEKDFDIR
AT1G56130  GLENGGYTVT LQFAEIQILG STSTTWKGLG RRRFDIYVQG RLVEKDFDVR
AT1G56140  GLENGGYTVT LQFAEIQILG STSNTWRGLG RRRFDIYVQG RLVEKDFDVR
AT1G07650  CLGNGNYTVN LHFAEIIFTD DNT--LYSLG KRLFDIYVQD QLVIKNFNIQ
AT1G29720  CLENGNYNVK LHFMEIQFSD KEV--YSRLG RRIFDVYVQG KLFLRDFNIN
AT1G29730  CFENGSYNVK LHFAEIQFSD VEP--YTKLA KRVFNIYIQG KLIWEDFSIR
AT1G29740  CFENGSYNVK LHFAEIQFSD EEP--FSRLA KRVFNIYVQG KLIWEDFSIR
AT1G29750  CLENGNYTIN LDFAEIRFTN DEN--YNRLG RRLFDIYIQE KLVAKDFNIM
AT1G53420  CLENGNYNVN LHFAEIMFNG NNN--YQSLG RRFFDIYIQR KLEVKDFNIA
AT1G53430  CLRRGSYKLQ LHFAEIMFSN DQT--FNSLG RRIFDIYVQG NLLERDFNIA
AT1G53440  CMRRGSYKVQ LYFAEIMFSN DQT--YSSLG RRLFDIYVQG ILLERDFNIA
AT3G14840  CLGKGNYTVN LHFAEIMFNE KNM--YSNLG RRYFDIYVQG KREVKDFNIV
AT2G22610  NLLPGEYLID FHFAEIINTN GPK------G IRVFNVYVQD E-KATEFDIF
AT1G72250  SLDPGDYFID LHFAEIEFTK GPP------G V--------- ---ISGLDLF

                  160        170        180        190        200
           ....|....| ....|....| ....|....| ....|....| ....|....|
X.laevis   DRVGHSTAHD EIIPISIKKG KLSVQGEVST FTGKLSVEFV KGYYDNPKVC
CORK1      KAANGSSIRV IQRVYKANVS ENYLEVHLFW AGKGTCCIPA QGTYG-PLVS
AT1G56120  RTAGGSSVRA VQREYKTNVS ENHLEVHLFW AGKGTCCIPI QGAYG-PLIA
AT1G56130  RTAGDSTVRA VQRVYKANVS ENHLEVHLFW AGKGTCCIPI QGAYG-PLIS
AT1G56140  RTAGDSTVRA VQREYKANVS QNHLEIHLFW AGKGTCCIPI QGAYG-PLIS
AT1G07650  EAARGSGKPI IKSFL-VNVT DHTLKIGLRW AGKGTTGIPI RGVYG-PMIS
AT1G29720  KEANGNMKPV IKEIN-ATVT NHMLEIRLYW AGKGTTLIPK RGNYG-PLIS
AT1G29730  EEANGTHKEV IREVN-TTVT DNTLEIRLYW AGKGTMIIPQ RGYYG-SLIS
AT1G29740  EEANGTHKEV IKEVN-TTVT DNTLEIRLYW AGKGTTIIPK RGNYG-SLIS
AT1G29750  DEAKGAQTPI IKPLT-AYVT NHFLTIRLSW AGKGTTRIPT RGVYG-PIIS
AT1G53420  KEAKDVGNVV IKTFP-VEIK DGKLEIRLYW AGRGTTVIPK ERVYG-PLIS
AT1G53430  ERAGGVGKPF IRQIDGVQVN GSTLEIHLQW TGKGTNVIPT RGVYG-PLIS
AT1G53440  QRAGGVGKPF LRQVDEVQVN GSTLEIHLKW TGKGTNVIPT RGVYG-PLIS
AT3G14840  DEAKGVGKAV VKKFP-VMVT NGKLEIRLQW AGKGTQAIPV RGVYG-PLIS
AT2G22610  SVVGANRPLL LVDLR----- -------VMV MDDGLIRVRF EGINGSPVVC
AT1G72250  SQVGANTPLV IEDLR----- -------MLV GREGELSIRL EGVTGAAILC
```

```
                     210        220        230        240        250
             ....|....| ....|....| ....|....| ....|....| ....|....|
X.laevis     ALFIMKGTAD DVPMLQPHPG LEKKEEEEEE EEEEGSTSKK QINKNRVQSG
CORK1        AI-------- ---------- ---------- ---------- ----------
AT1G56120    AV-------- ---------- ---------- ---------- ----------
AT1G56130    AV-------- ---------- ---------- ---------- ----------
AT1G56140    AV-------- ---------- ---------- ---------- ----------
AT1G07650    AI-------- ---------- ---------- ---------- ----------
AT1G29720    AI-------- ---------- ---------- ---------- ----------
AT1G29730    AV-------- ---------- ---------- ---------- ----------
AT1G29740    AI-------- ---------- ---------- ---------- ----------
AT1G29750    AI-------- ---------- ---------- ---------- ----------
AT1G53420    AI-------- ---------- ---------- ---------- ----------
AT1G53430    AI-------- ---------- ---------- ---------- ----------
AT1G53440    AI-------- ---------- ---------- ---------- ----------
AT3G14840    AV-------- ---------- ---------- ---------- ----------
AT2G22610    GI-------- ---------- ---------- ---------- ----------
AT1G72250    GI-------- ---------- ---------- ---------- ----------

                     260        270        280
             ....|....| ....|....| ....|....| ....|
X.laevis     PRTPNPYASD NSSLMFPILV AFGVFIPTLF CLCRL
CORK1        ---------- ---------- ---------- -----
AT1G56120    ---------- ---------- ---------- -----
AT1G56130    ---------- ---------- ---------- -----
AT1G56140    ---------- ---------- ---------- -----
AT1G07650    ---------- ---------- ---------- -----
AT1G29720    ---------- ---------- ---------- -----
AT1G29730    ---------- ---------- ---------- -----
AT1G29740    ---------- ---------- ---------- -----
AT1G29750    ---------- ---------- ---------- -----
AT1G53420    ---------- ---------- ---------- -----
AT1G53430    ---------- ---------- ---------- -----
AT1G53440    ---------- ---------- ---------- -----
AT3G14840    ---------- ---------- ---------- -----
AT2G22610    ---------- ---------- ---------- -----
AT1G72250    ---------- ---------- ---------- -----
```

Supplementary Figure S1. Alignment of malectin domains (MD) in *A. thaliana* and the malectin in *X. laevis*. Black shade indicates conserved amino acid residues over 90% threshold.

```
                    10         20         30         40         50
            ....|....| ....|....| ....|....| ....|....| ....|....|
X.laevis    ----MLSIRT VLGPLATILL TVLGPFGAHG SGLADKVIWA
CORK1       VNAGGESHVD ---------- ---------- ---------- ----------
AT1G29750   ---------- ---------- ---------- ---------- ----------
AT1G53430   ---------- ---------- ---------- ---------- ----------
AT1G56120   ---------- ---------- ---------- ---------- ----------
AT1G07650   ---------- ---------- ---------- ---------- ----------
AT1G29720   ---------- ---------- ---------- ---------- ----------
AT1G56140   ---------- ---------- ---------- ---------- ----------
AT1G56130   ---------- ---------- ---------- ---------- ----------
AT1G53440   ---------- ---------- ---------- ---------- ----------
AT1G29740   ---------- ---------- ---------- ---------- ----------
AT3G14840   ---------- ---------- ---------- ---------- ----------
AT1G53420   ---------- ---------- ---------- ---------- ----------
AT2G22610   ---------- ---------- ---------- ---------- ----------
AT1G29730   ---------- ---------- ---------- ---------- ----------
AT1G72250   ---------- ---------- ---------- ---------- ----------
AT1G51860   LDCGLVPKET -TYTEKSTNI TYKSDVDYID SGLVGKINDA YKTQF----Q
AT3G46240   IDCGTSLPGV D-----NNNL KWVGDQDFIT SG-DSATISS TTVEK-----
AT2G23200   VNCGSDSNVF YGGQTFVGDT NSSTNSVSFT NKGTEVINDQ SSVAP-----
AT2G14440   LYCGLPSNES -PYIEPLTNL TYISDVNFVR GGKTGNIKNN SDIDF---TS
AT1G30570   VDCGS-NATT EV-DGRTWVG DLSPNKSVTL QGFDAITAST -----SKGSS
AT3G05990   IDCGAS-SSS V-----IDGR QWQPDETFVS SG-TPKNVSD QVLDE-----
AT3G46420   VDCGLSPNEV SPYIEPFTGL QFTTDSNFIE TGKLGRIQAS LEPKY----R
AT1G25570   IDCGSPE-TS ----TDVFNR TWLPDQFYSG GSTAVVSEPL RFHLI-----
AT3G46370   ---------- ---------- ---------- ---------- ----------
AT3G46270   IDCGTTGSYV D-----SNNV TWVGDKGFVT TG-ESINIT- DVTTK-----
AT4G39110   IDCGSKSSSK TP-DGRVFKS DQET--IQYI EAKEDIQVSA ---PPSDKVA
AT1G07560   LDCGLQADES -PYTEPLTKL TFTSDADFIK SGKSGKIQNV PGMEY---I-
AT1G51820   VDCGLSLLES -PYDAPQTGL TYTSDADLVA SGKTGRLAKE FEPLV----D
AT1G24485   IDCGSSSSHI D-----ADNR TWVGDTDFVA TGLTSKFVPF SKFPA-----
AT5G39030   INCG-ETDVP FDNHGRTWTQ EEK---NILP KNSDN-ASFS SVVSYKEESG
AT3G46290   INCGS-PTNG TL-MGRIFLS DKLS--SKLL T---SSKEIL ----ASVGGN
AT5G61350   IDCGSSDETK LS-DGRNFKS DQQS--VAFL QTDEDIKTSV DSIPITDSNA
AT1G51805   VDCGLLPRDS -PYNALGTGL VYTSDVGLVS SGKTGKIAKE FEENN----S
AT5G59660   LDCGLPANEL SPYEESFTGL RFSSDEKFIR SGKNGRIREN -PQG----YA
AT5G39000   FNCG-DTSNN VDVSGRNWTA ENQ---KILS SNLVN-ASFT AQASYQE-SG
AT3G04690   LSCG-TSEAS ADQDKKKWEP DTK----FLK T--GN--SIH ATATYQDPSL
AT1G51790   IDCGLQPENS -SYTETSTDI KYVSDSSYTD TGTSYFVAPE NRQN----MK
AT5G28680   LSCG-ASEPA VDQDKKKWEP DTK----FLK T--PN--TVH APATYQDPSL
AT2G19190   IDCGI-PDDS -SYNDETTGI KYVSDSAFVD SGTTKRIAAQ FQSSG---FD
AT2G04300   LDCGLSPNEP -PYVDAATDL TYTTDNDFVQ SGKTGTIDKE LESTY----N
AT5G59680   LDCGLPANEP SPYTEPRTGL QFSSDAAFIQ SGKIGRIQAN -LEAD---FL
AT2G19210   IDCGI-PEDS -SYNDETTDI KYVSDAAFVE SGTIHSIDPE FQTSS---LE
AT1G51890   LDCGLVPTEI -TYVEKSTNI TYRSDATYID SGVPGKINEV YRTQF----Q
AT1G51880   LDCGLVPKNA -TYTEKTTNI TYKSDANYID SGLVGRISAE YKAQL----Q
AT5G59616   ---------- ---------- ---------- ---------- ----------
AT2G28970   LDCGFPIEES -PYSDPSTGL TFTSDSTFIQ TGESGRVDKE -LNKI---FR
AT5G54380   ISCGS-SQNI TF-QNRIFVP DSLH--SSLV LKIGNSSVAT ----STTSNN
AT5G48740   LSCGGSSYTA ------AYNI SWVSDNDYIE TGNTTTVTYA EGNST-----
AT2G28960   LDCGLPVNES -PYTDPRTGL TFSSDADFIL SGLRGEAGDD NT-----YIY
AT3G21340   LDCGSLPNEP -PYNDPSTGL TYSTDDGFVQ SGKTGRIQKA FESIF----S
AT3G46280   IDCGSTGSYV D-----SNNV TWVGDKGFVT NG-EPMKIP- DVVKK-----
AT3G46260   IDCGTTGSYV D-----SNNV TWVGDKGFVT TG-ESINIT- DVVKK-----
AT1G05700   IDCGI-PSGS -SYKDDTTGI NYVSDSSFVE TGVSKSIPFT AQ--------
AT3G46400   LDCGLSPNEQ SPYVELETGL QFLSDSSFIQ SGKIGRIDAS LESKY----P
AT5G38990   INCG-DTSNN MDYSGRNWTT ENP---KFMS SNAVDDASFT SSASYQE-SG
AT1G51800   LDCG-SPRET -SFREKTTNI TYISDANFIN TGVGGSIKQG YRTQF----Q
AT5G24010   INSGSNTNTS FF-TTRSFLS DSSEPGSSFL STDRSISISD -----TNPSP
AT2G28990   LDCGLPSDES -PYDDSFNGL TFTSDSTFIQ TGKIDSVDKD -LNIN---LS
AT4G20450   LDCGMPRNES -SYTDESTGL NFSSDADFIS SGKSGTIKTE DSDSGV-KYI
AT1G49100   LDCGLLPDGS -PYTNPSTGL TFTSDSSFIE SGKNGRVSKD SERNF----E
AT4G29450   IDCGI-PPYD -TPEDTMTNI NYVSDEAFIT TGVNFKVSEE YGYPKNPVLL
AT1G51840   VDCGLSPPES -PYNAPQTGL TYTSDTGLIN TGKTGRIAKD FEPFV----D
AT5G59670   LDCGLPANET SPYTETQTGL LFSSDATFIQ SGKTGRVQAN -QESK---FL
AT1G51850   VDCGLAPRES -PYNEAKTGL TYTSDDGLVN VGKPGRIAKE FEPLA----D
AT5G59700   INCGS-STNV TV-TSRVFIS DNLA--SNFL T---SPNEIL ----AASNRN
AT2G19230   IDCGI-PEDS -SYYDEKTDI KYISDAAFVE SGTIHSIDSK FQKKN---LE
AT1G51870   LDCGLVPKET -TYVETSTNI TYKSDANYTD SGLVGKINDA HKTLV----Q
AT3G46330   LDCGLPLNEP -PYIESETGI QFSSDENFIQ SGKTGRIPKN -LESE---NL
AT2G29000   LDCGLPAKES -PYTESTTSL VFTSDANFIS SGISTKLPKH DD-------Y
AT3G46350   LDCGLAPTEP SPYTEPVTTL QYSSDSNFIQ SGKLGRIDTS LQTFF----L
AT2G37050   LDCG----GA EPFTD-ELGL KWSPDNHLIY -GETANISSV NETRT-----
AT1G07550   LDCGLASNES -PYNEANSNL TYISDADFIQ GGKTGNVQKD LLMKL---R-
AT1G51810   LDCGLSIQGS -PYKESSTGL TYTSDDGFVQ SGKIGKITKE LESLY----K
AT4G29990   IDCGI-PDDS -SYTDEKTNM KYVSDLGFVE SGTSHSIVSD LQTTS---LE
AT5G59650   LDCGLPMTEP SSYTESVTGL RFSSDAEFIQ TGESGKIQAS -MEND---YL
AT1G28340   ISCGARKNVR ----TPPTYA LWFKDIAYTG GVPANATTPT -YITP-----
```

```
AT3G51550   LNCGGGASNL TDTDNRIWIS DVKS--KFLS SSSED--SKT SPALTQD-PS
AT2G21480   IDCGSKSSTK TP-EGRVFKS DSET--VQYI EAKDDIQVSA ---PPSDKLP
AT5G16900   LDCGLPSNEP -PYIEPVTGL VFSSDADHIP SGISGRIQKN -LEAV---HI
AT1G67720   IDCG----CS SNYTDPRTGL GWVSDSEIIK QGKPVTLANT NWNSM-----
AT3G19230   LNCGSS-SST N-----LNEI EYTPDEGFIS VG-NTTTIKQ KDLVP-----
AT1G51830   ---------- ---------- ---------- ---------- ----------
AT5G39020   FNCG-DTSNN VDNSGRNWTV ESR---QILS SNLVN-ASFT SEASYQK-AG
AT4G00300   VNCGSDVDST VD-NRR-FVG DASSSNVQFF SSEGSIALKG -----ENLPQ
AT4G29180   IDCGS-PPNI -NYVDTDTGI SYTWDAPFIN AGVNLNVSEE YGYPKNPVLP
AT1G51910   LDCGLIPKDT -TYTEQITNI TYISDADYID SGLTERISDS YKSQL----Q
AT3G46340   LDCGLPPNEV SPYIEPFTGL RFSSDSSFIQ SGKIGKVDKS FEATT----L
AT2G14510   LDCGLPSKES --YIEPSSNL TFISDVNFIR GGKTGNIQNN SRTNF---IF

                     60         70         80         90        100
            ....|....| ....|....| ....|....| ....|....| ....|....|
X.laevis    VHGIHYRKDP ---------- ----LEGRVG RASDYGMKLP ILRSNPE--
CORK1       D ---------- ---------- ---------- ----------
AT1G29750   ---------- ---------- ---------- ---------- ----------
AT1G53430   ---------- ---------- ---------- ---------- ----------
AT1G56120   ---------- ---------- ---------- ---------- ----------
AT1G07650   ---------- ---------- ---------- ---------- ----------
AT1G29720   ---------- ---------- ---------- ---------- ----------
AT1G56140   ---------- ---------- ---------- ---------- ----------
AT1G56130   ---------- ---------- ---------- ---------- ----------
AT1G53440   ---------- ---------- ---------- ---------- ----------
AT1G29740   ---------- ---------- ---------- ---------- ----------
AT3G14840   ---------- ---------- ---------- ---------- ----------
AT1G53420   ---------- ---------- ---------- ---------- ----------
AT2G22610   ---------- ---------- ---------- ---------- ----------
AT1G29730   ---------- ---------- ---------- ---------- ----------
AT1G72250   ---------- ---------- ---------- ---------- ----------
AT1G51860   QQVWAVRSFP -----VGQR- NCYNVNLT-- ANNKYLIRGT FVYGNYD-GL
AT3G46240   -SLTTLRYFP -----TGDS- NCYSNIPVT- KGGKVLVRTM FYYGNYDGKS
AT2G23200   EIYRTVRIFR ---------- HPSSYKFKLD SLGLHFVRLH FSVVFSRADL
AT2G14440   RPYKVLRYFP -----EGIR- NCYSLSVK-- QGTKYLIRTL FFYGNYD-GL
AT1G30570   VYAEIYKTAR -------VFD AVLNYTFEGI TQGNYFVRLH FSPFAIEN-H
AT3G05990   -ILFTVRSFP LSLDGTHHK- FCYV-MSVS- RGWKYMIRTT YYYGGVNGKG
AT3G46420   KSQTTLRYFP -----DGIR- NCYNLTVT-- QGTNYLIRAR AIYGNYD-GL
AT1G25570   -AEKTIRYFP ----LSFGKK NCY-VVPL-- PPGRYYLRTF TVYDNYD-GK
AT3G46370   ---------- -------MR- NCYNLSVH-- KETKYLIRVT SNYGNYD-GR
AT3G46270   -PINTLRYFP -----TGQT- NCYTNIPVT- KGRKTLVRTK YYYENYDDKF
AT4G39110   S--PIYLTAR -------IFR EEATYKFHLT RPGWHWVRLH FLAFPNDK-F
AT1G07560   KPYTVLRYFP -----DGVR- NCYTLIVI-- QGTNYLIVAM FTYGNYD-NL
AT1G51820   KPTLTLRYFP -----EGVR- NCYNLNVT-- SDTNYLIKAT FVYGNYD-GL
AT1G24485   -ELTTLRYFP -----TGET- NCYTNIPVE- KGGKVLVRTR FLYGDYDEES
AT5G39030   IPQVPYMTAR -------IFR SDFTYSFPVS PG-WKFLRLY FYPTSYKSGF
AT3G46290   SGSDIYHTAR -------VFT EVSSYKFSVT R-GRHWVRLY FNPFDYQN-F
AT5G61350   STLPLYLTAR -------IFA GKSTYSFYIS RPGRHWIRLH FYPLNHPL-Y
AT1G51805   TPNLTLRYFP -----DGAR- NCYNLNVS-- RDTNYMIKAT FVYGNYD-GH
AT5G59660   KP-------- ---------- ---------- ---------- FVYGNYD-GF
AT5G39000   VSQIPYMTAR -------IFR SEFTYSFPVT PG-SNFLRLY FYPTRYGSQF
AT3G04690   LSTVPYMTAR -------IFT APATYEIPIK GDKRHLLRLY FYPSTYTG-L
AT1G51790   QSMWSVRSFP -----EGIR- NCYTIAVN-- SSTKYLIRAD FMYGNYD-SR
AT5G28680   LSTVPYMTSR -------IFT APATYEIPVK GDKRHMLRLH FYPSTYTG-L
AT2G19190   RHLLNVRSFP -----QSKR- SCYDVPTPRG KGFKYLIRTR FMYGNYD-DL
AT2G04300   KPILQLRYFP -----EGVR- NCYTLNVT-- LGTNYLIRAS FVYGNYD-GL
AT5G59680   KPSTTMRYFP -----DGKR- NCYNLNVE-- KGRNHLIRAR FVYGNYD-GR
AT2G19210   KQFQNVRSFP -----EGNR- NCYDVKPPQG KGFKYLIRTR FMYGNYD-NL
AT1G51890   QQIWALRSFP -----EGQR- NCYNFSLT-- AKRKYLIRGT FIYGNYD-GL
AT1G51880   QQTWTVRSFP -----EGER- NCYNFNLT-- AKSRYLIRAT FTYGNYD-GL
AT5G59616   ---------- ---------- ---------- ---------- ----------
AT2G28970   KPYLTLRYFP -----EGKR- NCS------- ---------- ----------
AT5G54380   STNSIYQTAR -------VFS SLASYRFKIT SLGRHWIRLH FSPINNST-W
AT5G48740   -SSVPIRLFP -----DPQGR QCYKLPVR-K DLSSVLIRAT FVYRNYD-SQ
AT2G28960   RQYKDLRYFP -----DGIR- NCYNLKVE-- QGINYLIRAG FGYGNYD-GL
AT3G21340   KPSLKLRYFP -----DGFR- NCYTLNVT-- QDTNYLIKAV FVYGNYD-GL
AT3G46280   -PINTLRYFP -----TGQT- NCYTNIPVT- KGQKTLVRTK FYYENYDAKF
AT3G46260   -PINTLRYFP -----TGQT- NCYTNIPAT- KGRTTLVRTK FYYKNYDENY
AT1G05700   RQLQNLRSFP -----EGSR- NCYTLIPIQG KGKKYLIRAS FMYGNYD-GE
AT3G46400   RSQTTLRYFP -----DGIR- NCYNVNVY-- KGTNYLIRAT INYGNYD-GL
AT5G38990   IPQVPYLKAR -------IFR YDFTYSFPVS PG-WKFLRLY FYPTRYGSDF
AT1G51800   QQTWNLRSFP -----QGIR- NCYTLNLT-- IGDEYLIRAN FLHGGYD-DK
AT5G24010   DSPVLYNTAR -------VFP VGGSYKFQVT TKGTHFIRLH FAPFKASR-F
AT2G28990   KQYLTLRYFP -----EGKR- NCYSLDVK-- RGTTYLIVVS FVYGNYD-GL
AT4G20450   KPYKQLRYFP -----EGAR- NCYNLTVM-- QGTHYLIRAV FVYGNYD-LK
AT1G49100   KAFVTLRYFP -----DGER- NCYNLNVT-- QGTNYLIRAA FLYGNYD-GL
AT4G29450   STLAEVRAFP -----QGNR- NCYTLKLSQG KDHLYLIRAS FMYGNYD-GK
AT1G51840   KPALTMRYFP -----DGIR- NCYNLNVT-- RDTNYLIKAT FVYGNYD-GL
AT5G59670   KPYRTLRYFP -----EGVR- NCYNLSVF-- KERKYLIAAS FLYGNYD-GH
```

```
AT1G51850   KPTLTLRYFP -----EGVR- NCYNLNVT-- SDTNYLIKAT FVYGNYD-GL
AT5G59700   SNSDIYQTAR -------IFT GISKYRFSVA R-GRHWIRLH FNPFQYQN-F
AT2G19230   KQFQKVRSFP -----EGKK- NCYDVQPPQG KGFKYLIRTR FMYGNYD-NL
AT1G51870   QPLWALRSFP -----EGER- NCYNFNLT-- VNSTYLIRGT FLYGNYD-GL
AT3G46330   KQYATLRYFP -----DGIR- NCYDLRVE-- EGRNYLIRAT FFYGNFD-GL
AT2G29000   KPYNFLRYFP -----DGTR- HCYDLSVK-- QGTNYLIRAS FVYGNYD-GR
AT3G46350   KQQTTLRYFP -----DGIR- NCYNLTVK-- QGTNYLIRAR FTYGNYD-GR
AT2G37050   -QYTTLRHFP -----ADSRK YCYTLNVT-- SRNRYLIRAT FLYGNFDNSN
AT1G07550   KPYTVLRYFP -----DGIR- NCYSLNVK-- QDTNYLIRVM FRYGNYD-GL
AT1G51810   KPERTLRYFP -----DGVR- NCFSLNVT-- RGTKYLIKPT FLYGNYD-GR
AT4G29990   RQFQNVRSFP -----EGKR- NCYDIRPQQG KGFKYLIRTR FMYGNYD-GF
AT5G59650   KPYTRLRYFP -----EERR- NCYSLSVD-- KNRKYLIRAR FIYGNYD-GR
AT1G28340   -PLKTLRYFP ----ISEGPN NCYNIVRV-- PKGHYSVRIF FGLVDQP-SF
AT3G51550   VPEVPYMTAR -------VFR SPFTYTFPVA SG-RKFVRLY FYPNSYDG-L
AT2G21480   S--PIYLTAK -------IFR EEAIYKFHLT RPGWHWVRLH FFAFPNDK-F
AT5G16900   KPYLFLRYFP -----DGLR- NCYTLDVL-- QNRRYMIKAV FVYGNYD-GY
AT1G67720   -QYRRRRDFP -----TDNKK YCYRLSTK-- ERRRYIVRTT FLYGGLG-SE
AT3G19230   -ILSTLRYFP ---DKSSRK- HCYN-FPVA- KTSKYLIRTT YYYGNFDGKN
AT1G51830   ---------- ---------- ---------- ---------- ----------
AT5G39020   VSRIPYMKAR -------IFR SEFTYSFPVT PG-SIFLRLY FYPTQYKSGF
AT4G00300   NVPQIYRTAR -------IFA QQAKYKFNVN EKGTHMVRLH FNRLYSSR-I
AT4G29180   FPLADVRSFP -----QGNR- NCYTLTPSDG KGNLYLIRAS FMYGNYD-GK
AT1G51910   QQTWTLRSFP -----EGQR- NCYNFNLK-- ANLKYLIRGT FVYGNYD-GL
AT3G46340   KSYMTLRYFP -----DGKR- NCYNLIVK-- QGKTYMIRAT ALYGNYD-GL
AT2G14510   KPFKVLRYFP -----DGIR- NCYSLSVK-- QGTKYLIRTL FYYGNYD-GL

                      110        120        130        140        150
            ....|....| ....|....| ....|....| ....|....| ....|....|
X.laevis    QVLYQTERYN EDSFGYDIPI K--------- ---------- ------EEGE
CORK1       ---------- ---------- ---------- ---------- --------FN
AT1G29750   ---------- ---------- ---------- ---------- --------SC
AT1G53430   ---------- ---------- ---------- ---------- --------SS
AT1G56120   ---------- ---------- ---------- ---------- --------YN
AT1G07650   ---------- ---------- ---------- ---------- --------YK
AT1G29720   ---------- ---------- ---------- ---------- --------RF
AT1G56140   ---------- ---------- ---------- ---------- --------SD
AT1G56130   ---------- ---------- ---------- ---------- --------SD
AT1G53440   ---------- ---------- ---------- ---------- --------SS
AT1G29740   ---------- ---------- ---------- ---------- --------RS
AT3G14840   ---------- ---------- ---------- ---------- --------YG
AT1G53420   ---------- ---------- ---------- ---------- --------NA
AT2G22610   ---------- ---------- ---------- ---------- ----------
AT1G29730   ---------- ---------- ---------- ---------- --------RS
AT1G72250   ---------- ---------- ---------- ---------- ----------
AT1G51860   NQFPSFDLHI GPNKWSSVKI LGVTNT---- ---------- ----SMHEII
AT3G46240   STP-SFSVVF EGKHRGTLSI S--SAFEP-- ---------- ----YLLELI
AT2G23200   LTARFTVSAT SGSNHHLKSF SPQNLTN--- ---------- --TPRVEEFL
AT2G14440   NTSPRFDLFL GPNIWTSVDV QKVDG----- ---------- -GDGVIEEII
AT1G30570   NVNESSFSVF ADGLRLMLDI NIAGEIAHKN LILESTGHNA TASSLVKEFL
AT3G05990   TPPPVFDQIV DGTLWGIVNT T---ADYAD- ---------- -GLASYYEGV
AT3G46420   NIYPKFDLYI GPNFWVTIDL GKYVNG---- ---------- ----TWEEII
AT1G25570   SHSPSFDVSV EGTLVFSWRS PWPESLLRDG ---------- ----SYSDLF
AT3G46370   NEPPRFDLYL GPNFWVTIDL GKHVNGD--- ---------- ----TWKEII
AT3G46270   SPP-SFDIVY DGKHRDSVDI T-ESLLDDE- ---------- -DTFYFSEVI
AT4G39110   DLQQATFSVL TEKYVLLHNF KISNNNNDS- ---------- -QAAVQKEYL
AT1G07560   NTHPKFDLYL GPNIWTTVDL QRN------- ---------- -VNGTRAEII
AT1G51820   NVGPNFNLYL GPNLWTTVSS ND-------- ---------- ----TIEEII
AT1G24485   TYP-TFDVVY DGKHRYSVVT T------TF- ---------- -ETVTESEAI
AT5G39030   DAVNSFVSVT VNDFTLLQNF SADLTVKASI PE-------- SKSLIKEF-I
AT3G46290   KMGSAKFAVS SQSHVLLSDF TVTSS----- ---------- ---KVVKEYS
AT5G61350   NLTNSVFSVT TDTTVLLHDF SAG----DT- ---------- -SSIVFKEYL
AT1G51805   KDEPNFDLYL GPNLWATVSR SE-------- ---------- ----TVEEII
AT5G59660   DLKPKFDLYL GPNLWATVDL QTE------- ---------- -VNDWGNYTA
AT5G39000   NAVKSFFSVK VNGFTLLNNF SADLTVKASK PQ-------- TEFIIKEF-I
AT3G04690   NISNSYFTVE ANDVTLLSNF SAAITCQALT ---------- QAYLVKEYSL
AT1G51790   NEIPGFDLHL GPNKWDTVEL VSPLQ----- ---------- ---TVSKEII
AT5G28680   NILDSYFSVA ANDLTLLSNF SAAITCQALT ---------- QAYLVREYSL
AT2G19190   GRVPEFDLYL GVNFWDSVKL DDATT----- ---------- ---ILNKEII
AT2G04300   NKELEFDLYL GPNLWANVNT AVYLMNG--- ---------- ---VTTEEII
AT5G59680   DTGPKFDLYL GPNPWATIDL AKQ------- ---------- -VNGTRPEIM
AT2G19210   GKAPDFDLYL GFNIWDSVTI DNATT----- ---------- ---IVTKEII
AT1G51890   NQLPSFDLYI GPNKWTSVSI PGVRNG---- ---------- ----SVSEMI
AT1G51880   RQVPKFDIHI GPSKWTSVKL DGVGNG---- ---------- ----AVLEMI
AT5G59616   ---------- ---------- ---------- ---------- ----------
AT2G28970   ---------- ---------- ---------- ---------- ----------
AT5G54380   NLTSASITVV TEDFVLLNNF SFNNFN---- ---------- -GSYIFKEYT
AT5G48740   NSPPAFHVSL GRRITSTVDL RTNDP----- ---------- ----WIEELV
AT2G28960   NVYPKFDLHV GPNMWIAVDL EFG------- ---------- ----KDREII
AT3G21340   NNPPSFDLYL GPNLWVTVDM NGRTNG---- ---------- ----TIQEII
```

```
AT3G46280   SPP-SFDVIY DGKHRDSIVI T-ESLLNDE- ---------- -ETFYFSEVI
AT3G46260   SPP-SFDVVY DGKHRNSIAM TVDSLFSDE- ---------- -ETFHYSEVI
AT1G05700   NGSPEFDLFL GGNIWDTVLL SNGSS----- ---------- ---IVSKEVV
AT3G46400   NISPRFDLYI GPNFWVTIDL EKHVGGD--- ---------- ----TWEEII
AT5G38990   DAVKSFFSVN VNRFTLLHNF S----VKASI PE-------- SSSLIKEF-I
AT1G51800   -PSTQFELYL GPNLWSTVTT TNETEA---- ---------- ----SIFEMI
AT5G24010   NLRSAKFRVL INGFSVINSF STSS------ ---------- ---VVVKEFI
AT2G28990   NRDPNFDIHL GPNKWKRIDL DGE------- ---------- -KEGTREEII
AT4G20450   -QRPKFDLYL GPNFWTTINL QDPSGGFYYR IWL------- -QDGTVEEVI
AT1G49100   NTVPNFDLFI GPNKVTTVNF NATGGG---- ---------- ----VFVEII
AT4G29450   KALPEFDLYV NVNFWSTVKF KNASD----- ---------- ---QVTKEIL
AT1G51840   NVDPNFDLYL GPNLWTTVSS ND-------- ---------- ----TTEEII
AT5G59670   NIAPVFDLYL GPNLWAKIDL QD-------- ---------- -VNGTGEEIL
AT1G51850   NVGPNFDLYF GPNLWTTV-- ---------- ---------- ----------
AT5G59700   QMVSAKFSVS SETHVLLSDF TVSS------ ---------- ---RVMKEYS
AT2G19230   GKAPDFDLYL GVNLWDSVTL ENSTT----- ---------- ---IVTKEII
AT1G51870   NQSPSFDLHI GASKWTSVNI VGVTDT---- ---------- ----VMPEII
AT3G46330   NVSPEFDMHI GPNKWTTIDL QIV------- ---------- -PDGTVKEII
AT2G29000   NIMPRFDLYI GPNIWAVVSE LD----LYS- ---------- ----PEEEII
AT3G46350   NMSPTFDLYL GPNLWKRIDM TKLQNKVS-- ---------- ----TLEEIT
AT2G37050   NVYPKFDISL GATHWATIVI SETYI----- ---------- ---IETAELV
AT1G07550   NNSPRFDLYL GPNIWTTIDM GKS------- ---------- -GDGVLEEII
AT1G51810   NVIPDFDLYI GPNMWITVNT DN-------- ---------- ----TIKEIL
AT4G29990   SKTPEFDLYI GANLWESVVL INETA----- ---------- ---IMTKEII
AT5G59650   NSNPIFELHL GPNLWATIDL QKF------- ---------- -VNGTMEEIL
AT1G28340   DKEPLFDISI EGTQISSLKS GWSS--QDDQ ---------- ----VFAEAL
AT3G51550   NATNSLFSVS FGPYTLLKNF SASQTAEALT ---------- YAFIIKEF-V
AT2G21480   DLQQATFSVL TEKYVLLHNF KLSNDNNDS- ---------- -QATVQKEYL
AT5G16900   NDYPSFDLYL GPNKWVRVDL EGK------- ---------- -VNGSVEEII
AT1G67720   EAYPKFQLYL DATKWATVTI QEVSR----- ---------- ---VYVEELI
AT3G19230   NPP-VFDQII GGTKWSVVNT S---EDYAK- ---------- -GQSSYYEII
AT1G51830   ---------- ---------- ---------- ---------- ----------
AT5G39020   DAVNSFFSVK VNGFTLLRNF NADSTVQASI PL-------- SNSLIKEF-I
AT4G00300   DLNDALFHVT VNGHVVLRNF SGDSSS--D- ---------- -FESRVREFL
AT4G29180   NALPEFDLYV NVNFWTSVKL RNASE----- ---------- ---NVIKEIL
AT1G51910   NQMPKFDLHI GPNKWTSVIL EGVANA---- ---------- ----TIFEII
AT3G46340   NISPKFDLYI GANFWTTLDA GEYLSG---- ---------- ----VVEEVN
AT2G14510   NTSPRFDLFL GPNIWTSVDV LIADV----- ---------- -GDGVVEEIV

                      160        170        180        190        200
            ....|....| ....|....| ....|....| ....|....| ....|....|
X.laevis    YVLVLKFAEV YFAQ----SQ QKVFDVRVNG HTVVKD---- ----------
CORK1       FFVNCGGRDI RSS------S GALYEKDEGA LGPATFFVSK ----------
AT1G29750   LHVNCGGSDM YVKEK---KT KELYEGDGNV EGGAAKYFLK P---------
AT1G53430   LFINCGGSRL KIG------- KDTYTDDLNS RGQSTFSSVS ----------
AT1G56120   FSINCGGPEI RSV------S GALFEKEDAD LGPASFVVSA ----------
AT1G07650   LYINCGGGEV KVDKE---IT ---YQADDEP KG-ASMYVLG A---------
AT1G29720   LHINCGGEEV SIRNS---LG KITYQTDNSR QTNAASNQQ- ----------
AT1G56140   FSINCGGPEI RSV------T EAVFEREDED LGPASFVVSA ----------
AT1G56130   FSINCGGPEK RSV------T GALFEREDED FGPASFFVSA ----------
AT1G53440   LFINCGGNRL KVD------- KDEYADDLNK RGASTFSSVS ----------
AT1G29740   LHINCGGPDV TIENS---RG RFLYEGDNYG LTGSATNYY- ----------
AT3G14840   LHINCGGNEI TSN------- ETKYDADTWD TPG-YYDSK- ----------
AT1G53420   LHINCGGDEM SIN------- GTIYESDKYD RLESWYESR- ----------
AT2G22610   --------TI MFIN---AGG DDSKVLDSEL NISRDD---- ----YFEG--
AT1G29730   LHINCGGPDV TIENS---RG RFLYEGDNYG LTGSATNYY- ----------
AT1G72250   --------PV ISIN---SGS ISTDVTVEDV TFLKDE---- ----FFSG--
AT1G51860   HVVPQDSLEV CLVK---TGP TTPFISSLEV RPLNNE---- ----SYLTQS
AT3G46240   FSPAGGETSV CFVR--TSSS SNPFVSSIEV VDLDDGMY-- ----AELGPG
AT2G23200   LMMNSLFEFI RFVP---DHS SLALINAIEV FSAPDD---- ----LEIPSA
AT2G14440   HVTRCNILDI CLVK---TGT TTPMISAIEL RPLRYD---- ----TYTART
AT1G30570   LPTGPGKLVL SFIPE--KGS -FGFVNAIEI VSVDDKLFKE S--VTKVGGS
AT3G05990   FLAQGKSISV CVASNSYTTS -DPFISALEL VRLDGTLYNS ----TDFTTV
AT3G46420   YIPKSNMLDV CLVK---TGP STPLISSLVL RPLANA---- ----TYITQS
AT1G25570   AFIGDGELDL CFYS---IAT DPPIVGSLEV LQVDPS---- ----SYDA-D
AT3G46370   HIPKSNSLDV CLIK---TGT TTPIISTLEL RSLPKY---- ----SYNAIS
AT3G46270   FAPASENISV CLLR--TSPS DNPFISSIEV YSFDDGMY-- ----KDLGPE
AT4G39110   VNMTDAQFAL RFRPM--KSS -AAFINAIEV VSAPDELISD S--GTALFPV
AT1G07560   HIPRSTSLQI CLVK---TGT TTPLISALEL RPLRNN---- ----TYIPQS
AT1G51820   LVTRSNSLQV CLVK---TGI SIPFINMLEL RPMKKN---- ----MYVTQS
AT1G24485   FIPENGNISV CFFR--TLSS KTPFVSTIEV RRLDDSMY-- ----TDLGPK
AT5G39030   VPVY-LTLNL TFRPS---NN SLAFVNGIEI VSMPDRFYSK GGFDDLITNV
AT3G46290   LNVTTNDLVL TFTPS--SGS -FAFVNAIEV ISIPDTLITG S--PRFVGNP
AT5G61350   I-YAAEKLSL YFKPH--KGS -TAFINAVEI VSVPDELVPD S--ASSVPQA
AT1G51805   HVTKSDSLQV CLAK---TGD FIPFINILEL RPLKKN---- ----VYVTES
AT5G59660   NIGFG----- ---------- ---------- -IMGNG---- ----SYITKS
AT5G39000   IPVY-QTLNL TFTPS---LD SLAFVNGIEI VSIPNRFYSK GGFDDVITNV
AT3G04690   APTDKDVLSI KFTPSDKYRD AFAFINGIEV IQMPELFDTA ----ALVGFT
AT1G51790   YYVLTDTIQV CLVN---TGN GTPFISVLEL RQLPNS---- ----SYAAQS
```

```
AT5G28680  APSEKDVLSI IFTPSDKHPK AFAFINGIEV IPMPELFDTA ----SLVGFS
AT2G19190  TIPLLDNVQV CVVD---KNA GTPFLSVLEI RLLLNT---- ----TYETPY
AT2G04300  HSTKSKVLQV CLIK---TGE SIPIINSLEL RPLIND---- ----TYNTQS
AT5G59680  HIPTSNKLQV CLVK---TGE TTPLISVLEV RPMGSG---- ----TYLTKS
AT2G19210  HTLRSDHVHV CLVD---KNR GTPFLSALEI RLLKSN---- ----TYETPY
AT1G51890  HVLRQDHLQI CLVK---TGE TTPFISSLEL RPLNNN---- ----TYVTKS
AT1G51880  HVLTQDRLQI CLVK---TGK GIPFISSLEL RPLNNN---- ----TYLTQS
AT5G59616  ---------- ---------- ---------- ---------- ----------
AT2G28970  ---------- ---------- ---------- --LRN----- ----------
AT5G54380  VNVTSEFLTL SFIPS--NNS -VVFVNAIEV VSVPDNLIPD Q--ALALNPS
AT5G48740  WPVNNDSLLL CLLAVK--GR GIPVISSLEV RPLPLG---- ----SYKYSL
AT2G28960  YMTTSNLLQI CLVK---TGS TIPMISTLEL RPLRND---- ----SYLTQF
AT3G21340  HKTISKSLQV CLVK---TGT SSPMINTLEL RPLKNN---- ----TYNTQS
AT3G46280  YVPENKNISV CLLR--TSPS DNPFISSIEV YSLDTGMY-- ----DDLGPN
AT3G46260  FAPANENISV CLVR--TSPS DNPFISSIEV YRFDAGMY-- ----DDLGPE
AT1G05700  YLSQSENIFV CLGN---KGK GTPFISTLEL RFLGNDNT-- ----TYDSPN
AT3G46400  HIPKSNSLDV CLIK---TGT STPIISVLEL RSLPNN---- ----TYITES
AT5G38990  VPVN-QTLDL TFTPS---PN SLAFVNGIEI ISMPDRFYSK GGFDDVVRNV
AT1G51800  HILTTDRLQI CLVK---TGN ATPFISALEL RKLMNT---- ----TYLTRQ
AT5G24010  LKIDDPVLEI SFLPF--KAS GFGFVNAVEV FSAPKDYIMD Q--GTKLVIP
AT2G28990  HKARSNSLDI CLVK---TGE TLPIISAIEI RPLRNN---- ----TYVTQS
AT4G20450  HMPKSNNLDI CLVK---TGT TTPFISSLEL RPLRDD---- ----TYTTTT
AT1G49100  HMSRSTPLDI CLVK---TGT TTPMISTLEL RPLRSD---- ----TYISAI
AT4G29450  SFAESDTIYV CLVN---KGK GTPFISGLEL RPVNSS---- ----IYGTEF
AT1G51840  HVTKFNSLQI CLVK---TGI SIPFINVLEL RPLKKN---- ----VYATQS
AT5G59670  HIPTSNSLQI CLVQ---TGE TTPLISSLEL RPMRTG---- ----SYTTVS
AT1G51850  ---------- CLIK---TGI SIPFINVLEL RPMKKN---- ----MYVTQG
AT5G59700  LNVATDHLEL TFTPS--GDS -FAFLNALEV VSVPDTLFSG D--PSFAGSP
AT2G19230  YTLRSDKVHV CLVD---KER GTPFLSVLEL RLLKNN---- ----IYETAS
AT1G51870  HVLTQKRLQV CLVK---TGK TTPFISSLEL RPLINN---- ----IYIAES
AT3G46330  HIPRSNSLQI CLVK---TGA TIPMISALEL RPLAND---- ----TYIAKS
AT2G29000  HMTKSTSLQI CLVK---TGP TTPFISTLEL RPLRND---- ----NYITQS
AT3G46350  YIPLSNSLDV CLVK---TNT TIPFISALEL RPLPSN---- ----SYITTA
AT2G37050  FLASSPTVSV CLSN---ATT GQPFISTLEL RQLSGS-M-- ----YGSMLS
AT1G07550  HITRSNILDI CLVK---TGT STPMISSIEL RPLLYD---- ----TYIAQT
AT1G51810  HVSKSNTLQV CLVK---TGN SIPYINTLEL RPLADD---- ----IYTNES
AT4G29990  YTPPSDHIHV CLVD---KNR GTPFLSVLEI RFLKND---- ----TYDTPY
AT5G59650  HTPTSNSLNV CLVK---TGT TTPLISALEL RPLGNN---- ----SYLT-D
AT1G28340  IFLLGGTATI CFHS---TGH GDPAILSIEI LQVDDK---- ----AYSFGE
AT3G51550  VNVEGGTLNM TFTPESAPSN AYAFVNGIEV TSMPDMYSST ---DGTLTMV
AT2G21480  LNMTDAQFAL RFKPM--KGS -AAFINGIEL VSAPDELISD A--GTSLFPV
AT5G16900  HIPSSNSLQI CLVK---TGN SLPFISALEL RLLRND---- ----TYVVQD
AT1G67720  VRATSSYVDV CVCC---AIT GSPFMSTLEL RPLNLS-M-- ----YATDY-
AT3G19230  VGVPGNRLSV CLAKNAHTLS SSPFISSLDV QSLEDTMYNS ----TDLGSY
AT1G51830  ---------- ---------- ---------- ---------- ----------
AT5G39020  IPVH-QTLNL TFTPS---KN LLAFVNGIEI VSMPDRFYSK GGFDNVLRNV
AT4G00300  IWVDVGEVVI RFVPS--KDS NFAFVNAIEV ISAPKDLIGD V--ATS-VSH
AT4G29180  SFAESDTIYV CLVN---KGK GTPFISALEL RPMNSS---- ----IYGTEF
AT1G51910  HVLTQDRLQV CLVK---TGQ TTPFISSLEL RPLNND---- ----TYVTQG
AT3G46340  YIPRSNSLDV CLVK---TDT STPFLSLLEL RPLDND---- ----SYLTGS
AT2G14510  HVTRSNILDI CLVK---TGT STPMISAIEL RPLRYD---- ----TYTART

                    210        220        230        240        250
           ....|....| ....|....| ....|....| ....|....| ....|....|
X.laevis   ---------- ---------- ---------- --LDIFDRVG HST-------
CORK1      ---------- ---------- ---------- ------TQRW AVSNVGL--F
AT1G29750  ---------- ---------- ---------- ------DANW GFSSTGD--F
AT1G53430  ---------- ---------- ---------- -------ERW GYSSSGV--W
AT1G56120  ---------- ---------- ---------- ------AKRW AASSVGN--F
AT1G07650  ---------- ---------- ---------- ------NKRW ALSSTGN--F
AT1G29720  ---------- ---------- ---------- ------FDYW GVSNTGD--F
AT1G56140  ---------- ---------- ---------- ------GQRW AASSVGL--F
AT1G56130  ---------- ---------- ---------- ------GQRW AASSVGL--F
AT1G53440  ---------- ---------- ---------- -------ERW GYSSSGA--W
AT1G29740  ---------- ---------- ---------- ------GKNW GFSNTGD--F
AT3G14840  ---------- ---------- ---------- -------NGW VSSNTGN--F
AT1G53420  ---------- ---------- ---------- -------NGW FSNNVGV--F
AT2G22610  ---------- ---------- ---------- ---------- ----------
AT1G29730  ---------- ---------- ---------- ------RKNW GYSNTGD--F
AT1G72250  ---------- ---------- ---------- ---------- ----------
AT1G51860  GS---LMLFA RVYFPSSSSS FIR------- YDEDIHDRVW NSF-------
AT3G46240  E--------- ---------- ---------- ------GRFW LP--------
AT2G23200  SD----KNLH TIYRLNVGGE KITP------ -DNDTLGRTW LPDDDDFLYR
AT2G14440  GS---LKKIL HFYF-TNSGK EVR------- YPEDVYDRVW IPH-------
AT1G30570  E---VELGLG GRGIETMYRL NVGGPKLGPS KDL-KLYRTW ETDLSYM--V
AT3G05990  GM-----SLV ARHAFGYSGP IIR------- FPDDQFDRFW EPYS------
AT3G46420  GW---LKTYV RVYL-SDSND VIR------- YPDDVYDRIW GSY-------
AT1G25570  GTGQNVLLVN YGRLSCGSDQ WGPGFT--NH TDNFG--RSW QSDEDFRS--
AT3G46370  GS---LKSTL RAFL-SESTE VIR------- YPNDFYDRMW VPH-------
```

```
AT3G46270   EG-----LIL YQRITYGAKK LIS------- YPLDPFGRLW SPSA------
AT4G39110   I---GFSGLS DYAYQSVYRV NVGGPLIMPQ ND--TLGRTW IPDKEFL--K
AT1G07560   GS---LKTLF RVHL-TDSKE TVR------- YPEDVHDRLW SPF-------
AT1G51820   GS---LKYLF RGYI-SNSST RIR------- FPDDVYDRKW YP--------
AT1G24485   EG-----FIL QQRIAYGAQE LVR------- FPYDPYDRIW MP--------
AT5G39030   GSLIDFEIDN STASETVHRL NVGGHMVDEV NDSG-MFRRW LSDDYEF--L
AT3G46290   A---QFPDMS MQGLETIHRV NMGGPLVASN ND--TLTRTW VPDSEFL--L
AT5G61350   P---DFKGLS SFSLEILHRI NIGGDLISPK ID--PLSRTW LSDKPYN--T
AT1G51805   GS---LKLLF RKYF-SDSGQ TIR------- YPDDIYDRVW HA--------
AT5G59660   GS---LNLLS RTYL-SKSGS DLR------- YMKDVYDRTW VSYG------
AT5G39000   GSSVDFHIEN STAFETVYRL NVGG---KTV GDSG-MFRRW VSDDEII--L
AT3G04690   DQTMDAKTAN ---LQSMFRL NVGGQDIPGS QDSGGLTRTW YNDAPYI--F
AT1G51790   E---SLQLFQ RLDFGSTTNL TVR------- YPNDVFDRIW FPA-------
AT5G28680   DQTSDTKTAN ---LQTMFRL NVGGQDIPGS QDSGGLTRTW YNDAPYI--F
AT2G19190   D---ALTLLR RLDYSKTGKL PSR------- YKDDIYDRIW TPR-------
AT2G04300   GS---LKYLF RNYF-STSRR IIR------- YPNDVNDRHW YP--------
AT5G59680   GS---LKLYY REYF-SKSDS SLR------- YPDDIYDRQW TSF-------
AT2G19210   D---SLILFK RWDLGGLGAL PVR------- YKDDVFDRIW IP--------
AT1G51890   GS---LIVVA RLYF-SPTPP FLR------- YDEDVHDRIW IPF-------
AT1G51880   GS---LIGFA RVFF-SATPT FIR------- YDEDIHDRVW VRQ-------
AT5G59616   ---------- ---------- ---------- ---------- ----------
AT2G28970   --------SF RVHC-STSDS EIR------- YDDDSYDRVW YPF-------
AT5G54380   T---PFSGLS LLAFETVYRL NMGGPLLTSQ ND--TLGRQW DNDAEYL--H
AT5G48740   EGSPDIILRR SYRINSGYTN GTIR------ YPSDPFDRIW DPDQ------
AT2G28960   GP---LDLIY RRAY-SSNST GFIR------ YPDDIFDRKW DR--------
AT3G21340   GS---LKYFF RYYF-SGSGQ NIR------- YPDDVNDRKW YP--------
AT3G46280   EG-----LIL HDRIAYGAKE LIS------- YPLDPYGRVW LALG------
AT3G46260   EG-----FIL YKRNAYGATK LIS------- YPLDPYGRLW SPKG------
AT1G05700   G---ALFFSR RWDLRSLMGS PVR------- YDDDVYDRIW IP--------
AT3G46400   GS---LKSIL RSYL-SVSTK VIR------- YPDDFYDRKW VPY-------
AT5G38990   GRDVDFEIDN STAFETVYRV NVGGKVVGDV GDSG-MFRRW LSDEGFL--L
AT1G51800   GS---LQTFI RADVGATVNQ GYR------- YGIDVFDRVW TPY-------
AT5G24010   NSAQIFSNLS SQVLETVHRI NVGGSKLTPF ND--TLWRTW VVDDNYL--L
AT2G28990   GS---LMMSF RVYL-SNSDA SIR------- YADDVHDRIW SPF-------
AT4G20450   GS---LKLIS RWYF-RKPFP TLESII--R- HPDDVHDRLW DVY-------
AT1G49100   GSS--LLLYF RGYL-NDSGV VLR------- YPDDVNDRRW FP--------
AT4G29450   GRNVSLVLYR RWDIGYLNG- TGR------- YQDDRFDRIW SPY-------
AT1G51840   GS---LKYLF RMYV-SNSSR RIR------- ---------- ----------
AT5G59670   GS---LKTYR RLYF-KKSGS RLR------- YSKDVYDRSW FPR-------
AT1G51850   ES---LNYLF RVYI-SNSST RIR------- FPDDVYDRKW YP--------
AT5G59700   G---KFQGLS WQALETVYRV NMGGPRVTPS ND--TLSRIW EPDSEFL--V
AT2G19230   D---SLMLYR RWDLGATGDL PAR------- YKDDIFDRFW MP--------
AT1G51870   GS---MVLQN RVYFPSDSTS IVR------- YDEDIHDRVW NPV-------
AT3G46330   GS---LKYYF RMYL-SNATV LLR------- YPKDVYDRSW VPY-------
AT2G29000   GS---LKLMQ RMCM-TETVS TLR------- YPDDVYDRLW YT--------
AT3G46350   GS---LRTFV RFCF-SNSVE DIR------- FPMDVHDRMW ESY-------
AT2G37050   EDRFYLSVAA RINFGAESEA SVR------- YPDDPYDRIW ESDLQKKPNY
AT1G07550   GS---LRNYN RFYF-TDSNN YIR------- YPQDVHDRIW VPL-------
AT1G51810   GS---LNYLF RVYY-SNLKG YIE------- YPDDVHDRIW KQ--------
AT4G29990   E---ALMLGR RWDFGTATNL QIR------- YKDDFYDRIW MP--------
AT5G59650   GS---LNLFV RIYL-NKTDG FLR------- YPDDIYDRRW HNY-------
AT1G28340   GWGQGVILRT ATRLTCGTGK SRFDED--YR GDHWGGDRFW NRMRSFG---
AT3G51550   GSSGSVTIDN STALENVYRL NVGGNDISPS ADTG-LYRSW YDDQPYI--F
AT2G21480   N---GFSGLS DYAYQSVYRV NVGGPLITPQ ND--TLGRTW TPDKEYL--K
AT5G16900   VS---LKHLF RRYY-RQSDR LIR------- YPDDVYDRVW SPF-------
AT1G67720   EDNFFLKVAA RVNFGAPNMD ALR------- YPDDPYDRIW ESDINKRPNY
AT3G19230   KL-----SLI ARNSFGGDGE IIS------- YPDDKYNRLW QPFS------
AT1G51830   ---------- -FFI-NDC-- -VR------- FPDDVYDRKW YP--------
AT5G39020   SSDVDFQIDN STAFESVHRL NVGGQIVNEV DDSG-MFRRW LSDD-----S
AT4G00300   DGTEKFNGLA KQAMEVVYRV NVGGRKVTPF ND--TLWRTW VTDEGFL--K
AT4G29180   GRNVSLVLYQ RWDTGYLNG- TGR------- YQKDTYDRIW SPY-------
AT1G51910   GS---LMSFA RIYF-PKTAY FLR------- YSDDLYDRVW VPF-------
AT3G46340   GS---LKTFR RYYL-SNSES VIA------- YPEDVKDRIW EPT-------
AT2G14510   GS---LKSMA HFYF-TNSDE AIR------- YPEDVYDRVW MPY-------

                   260        270        280        290        300
            ....|....| ....|....| ....|....| ....|....| ....|....|
X.laevis    ---AHDEIIP ISIKKGKLSV QGEVSTFTGK LSVEFVKGYY DN--------
CORK1       TGSNS--NQY IALSATQ--- -FANTSDSE- LFQSARL--- -------SAS
AT1G29750   MDDNN----- -FQNTRF-TM FVPASNQSD- LYKSARI--- -------APV
AT1G53430   LGKED--AGY LATDRFN--- -LINGSTPE- YYKTARL--- -------SPQ
AT1G56120   AGSSN--NIY IATSLAQ--- -FINTMDSE- LFQSARL--- -------SAS
AT1G07650   MDNDDDADEY TVQNTSR-LS VNASSPSFG- LYRTARV--- -------SPL
AT1G29720   TDDNSDHDEY YTSTNL---- -TLSGDYPD- LYKTARR--- -------SAL
AT1G56140   AGSSN--NIY ISTSQSQ--- -FVNTLDSE- LFQSARL--- -------SAS
AT1G56130   AGSSN--NIY IATSQSQ--- -FVNTLDSE- LFQSARL--- -------SAS
AT1G53440   LGNDG--ATY LATDTFN--- -LINESTPE- YYKTARL--- -------ASQ
AT1G29740   MDDAITEDTY TVSSES---- -AVSAKYPD- LYQNARR--- -------SPL
AT3G14840   LDDDRTNNGK SKWSNSSELK ITNSSIDFR- LYTQARL--- -------SAI
```

```
AT1G53420   VDDKHVPERV TIESNSSELN V----VDFG- LYTQARI--- -------SAI
AT2G22610   -------GDV LRTEESIVEA GDFP-----F IYQSARVGN- ----------
AT1G29730   MDDAITEDTY TVSSES---- -AVSAKYPD- LYQNARR--- -------SPL
AT1G72250   -------GES I-TTDAVVGN EDEI-----L LYQTARLGN- ----------
AT1G51860   ---TDDETVW ISTDLPIDTS -NS-YDMPQS VMKTAAVPKN AS--------
AT3G46240   --SEINILVT GIQSTAVSID TSGASNKPPE SVLRNSWTG- --E-------
AT2G23200   KDSARNINST QTPNYVGGLS SATDSTAPDF VYKTAKAMNR SSNE-QVGML
AT2G14440   ---SQPEWTQ INTTRNVSGF SD-GYNPPQD VIKTASIPTN VS--------
AT1G30570   IENAGVEVKN S-SNITYALA DDS-PVAPLL VYETARMMSN TEV--LEKRF
AT3G05990   ----LNSTVP ----NNRKLE VSGFWNLPPS RIFNTDLRAT QVQ-------
AT3G46420   ---FEPEWKK ISTTLGVN-S SSG-FLPPLK ALMTAASPAN AS--------
AT1G25570   -EDSRSVARS LSTLEKIKGV DQAPNYFPMK LYQTAVTVSG GG--------
AT3G46370   ---FETEWKQ ISTNLKVN-S SNG-YLLPQD VLMTAAIPVN TS--------
AT3G46270   --SGDNTALT DLSTSAPSID ITGASNKPPE IVMSKALSG- --D-------
AT4G39110   DENLAKDVKT TPSAIKYPP- EVTPLIAPQT VYATAVEMAN SLT--IDPNF
AT1G07560   ---FMPEWRL LRTSLTVNTS DDNGYDIPED VVVTAATPAN VS--------
AT1G51820   -LFD-DSWTQ VTTNLKVNTS IT--YELPQS VMAKAATPIK AN--------
AT1G24485   ----ASVFAS HLTSSATSID TTGADNRPPE IILRTSWSQ- --K-------
AT5G39030   IG--GVSPYM P-DVNISYTE KTPAYVAPAY VYSTCRMMGN AQDTYLNLNF
AT3G46290   EKN-LAKSMS KFSTVNFVPG YATEDSAPRT VYGSCTEMNS ADN--PNSIF
AT5G61350   FPEGSRNVTV DPSTITYPDG GATALIAPNP VYATAEEMAD AQT--SQPNF
AT1G51805   -SFLENNWAQ VSTTLGVNVT DN--YDLSQD VMATGATPLN DS--------
AT5G59660   -ASFRTGWTQ IYTALEVNNS NN--YAPPKD ALRNAATPTN AS--------
AT5G39000   SESSGISPIV P-DIKINYTE KTPSYVAPDD VYATSRSMGN ADHPEQNLNF
AT3G04690   SAGLGVTLQA SNNFRINY-Q NMPVSIAPAD IYKTARSQGP N--GDINLKS
AT1G51790   --TPNGTKPL SDPSTSLTSN STGNFRLPQV VMRTGIVPDN PR--------
AT5G28680   SAGLGVTLQA SNNFRIDY-Q KMPVSTAPAD VYKTARSQGP N--GDINMKS
AT2G19190   --IVSSEYKI LNTSLTVDQF LNNGYQPAST VMSTAETARN ES--------
AT2G04300   -FFDEDAWTE LTTNLNVNSS NG--YDPPKF VMASASTPIS KN--------
AT5G59680   --FDT-EWTQ INTTSDVGNS ND--YKPPKV ALTTAAIPTN AS--------
AT2G19210   --LRFPKYTI FNASLTIDSN NNEGFQPARF VMNTATSPED LS--------
AT1G51890   ---LDNKNSL LSTELSVDTS -NF-YNVPQT VAKTAAVPLN AT--------
AT1G51880   ---FGNGLKS ISTDLLVDTS -NP-YDVPQA VAKTACVPSN AS--------
AT5G59616   ---------- ---------- ---------- ---------- ----------
AT2G28970   ---FSSSFSY ITTSLNINNS DT--FEIPKA ALKSAATPKN AS--------
AT5G54380   VNSSVLVVTA NPSSIKYSP- SVTQETAPNM VYATADTMGD ANV--ASPSF
AT5G48740   -SYSPFHASW SFNGLTKLNS FNITENPPAS VLKTARILAR KE--------
AT2G28960   ---YNEFETD VNTTLNVR-S SSP-FQVPEA VSRMGITPEN AS--------
AT3G21340   -FFDAKEWTE LTTNLNINSS NG--YAPPEV VMASASTPIS TF--------
AT3G46280   --SQD-STLT DLTTSAPSID ITGASNKPPE IVMSKALSG- --V-------
AT3G46260   --SQDYPGLI DLTTSAPSID ITGALNKPPE IVMTKAMSG- --D-------
AT1G05700   --RNFGYCRE INTSLPVTS- DNNSYSLSSL VMSTAMTPIN TT--------
AT3G46400   ---FESEWRQ ISTILKVNNT ING-FLAPQE VLMTAAVPSN AS--------
AT5G38990   GINSGAIPNI T-GVKINYTD KTPAYVAPED VYTTCRLMGN KDSPELNLNF
AT1G51800   ---NFGNWSQ ISTNQSVNIN -ND-YQPPEI AMVTASVPTD PD--------
AT5G24010   LR-AAARRAW TTHSPNYQNG GATREIAPDN VYMTAQEMDR DNQE-LQARF
AT2G28990   ---NGSSHTH ITTDLNINNS NA--YEIPKN ILQTAAIPRN AS--------
AT4G20450   --HADEEWTD INTTTPVNTT VNA-FDLPQA IISKASIPQV AS--------
AT1G49100   -FSY-KEWKI VTTTLNVNTS NG--FDLPQG AMASAATRVN DN--------
AT4G29450   --SSNISWNS IITSGYIDVF QNG-YCPPDE VIKTAAAPEN VD--------
AT1G51840   ---------- ---------- ---------- ---------- ----------
AT5G59670   --FMD-EWTQ ISTALGVINT NI--YQPPED ALKNAATPTD AS--------
AT1G51850   -YFD-NSWTQ VTTTLDVNTS LT--YELPQS VMAKAATPIK AN--------
AT5G59700   EKN-LVKSVS KIASVDYVPG FATEETAPRT VYGTCTEMNS ADN--PSSNF
AT2G19230   --LMFPNFLI LNTSLMIDPT SSNGFLPPSV VMSTAVAPMN SSI-------
AT1G51870   ---SDDDSSS ISTDLQVQTN -NL-YDVPQF VMKTAAIPKD AS--------
AT3G46330   ---IQPEWNQ ISTTSNVSNK NH--YDPPQV ALKMAATPTN LD--------
AT2G29000   --DGIYETKA VKTALSVN-S TNP-FELPQV IIRSAATPVN SS--------
AT3G46350   ---FDDDWTQ ISTSLTVN-T SDS-FRLPQA ALITAATPAK DG--------
AT2G37050   LVDVAAGTVR VSTTLPIESR VDD--RPPQK VMQTAVVGTN GS--------
AT1G07550   ---ILPEWTH INTSHHVIDS ID-GYDPPQD VLRTGAMPAN AS--------
AT1G51810   -ILPYQDWQI LTTNLQINVS ND--YDLPQR VMKTAVTPIK AST-------
AT4G29990   --YKSPYQKT LNTSLTIDET NHNGFRPASI VMRSAIAPGN ES--------
AT5G59650   --FMVDDWTQ IFTTLEVTND NN--YEPPKK ALAAAATPSN AS--------
AT1G28340   ----KSADSP RSTEETIKKA SVSPNFYPEG LYQSALVSTD DQ--------
AT3G51550   GAGLGIPETA DPNMTIKYPT GTPTYVAPVD VYSTARSMGP T--AQINLNY
AT2G21480   DENLAKDVKT NPTAIIYPP- GVTPLIAPQT VYATGAEMAD SQT--IDPNF
AT5G16900   ---FLPEWTQ ITTSLDVNNS NN--YEPPKA ALTSAATPGD NG--------
AT1G67720   LVGVAPGTTR INTSKTINTL TRE--YPPMK VMQTAVVGTQ GL--------
AT3G19230   --DQKHLTVT ----SRSRIN PSNFWNIPPA EAFVEGFTAS KGK-------
AT1G51830   -IFQ-NSWTQ VTTNLNVNIS TI--YELPQS VMSTAATPLN AN--------
AT5G39020   FGNSGSIVNV P-GVKINYTE KTPAYVAPYD VYATSRLMGN S----SNLMF
AT4G00300   TGDGSSEKSY FTGRIKYRRG GASREVGPDN VYNTARVGKR SN-----GLV
AT4G29180   --SP-VSWNT TMTTGYIDIF QSG-YRPPDE VIKTAASPKS DD--------
AT1G51910   ---SQNETVS LSTNLPVDTS SNS-YNVPQN VANSAIIPAE AT--------
AT3G46340   ---FDSEWKQ IWTTLKPN-N SNG-YLVPKN VLMTAAIPAN DS--------
AT2G14510   ---SQPEWTQ INTTRNVSGF SD-GYNPPQG VIQTASIPTN GS--------
```

```
                        310       320       330       340       350
              ....|....| ....|....| ....|....| ....|....| ....|....|
X.laevis      PKVCALFIMK GTADDVPMLQ PHPGLEKKEE EE-------- ----------
CORK1         SLRYYGLGLE --NGGYSVTV QFAEIQIQGS NT---WKSLG RRIFDIYVQG
AT1G29750     SLTYFHACLE --NGNYTINL DFAEIRFTND EN---YNRLG RRLFDIYIQE
AT1G53430     SLKYYGLCLR --RGSYKLQL HFAEIMFSND QT---FNSLG RRIFDIYVQG
AT1G56120     SLRYYGLGLE --NGGYTVTL QFAEVQIEGS NS---WKGIG RRRFNIYVQG
AT1G07650     SLTYYGICLG --NGNYTVNL HFAEIIFTDD NT---LYSLG KRLFDIYVQD
AT1G29720     SLVYYAFCLE --NGNYNVKL HFMEIQFSDK EV---YSRLG RRIFDVYVQG
AT1G56140     SLRYYGLGLE --NGGYTVTL QFAEIQILGS TSNT-WRGLG RRRFDIYVQG
AT1G56130     SVRYYGLGLE --NGGYTVTL QFAEIQILGS TSTT-WKGLG RRRFDIYVQG
AT1G53440     SLKYYGLCMR --RGSYKVQL YFAEIMFSND QT---YSSLG RRLFDIYVQG
AT1G29740     SLAYFAICFE --NGSYNVKL HFAEIQFSDE EP---FSRLA KRVFNIYVQG
AT3G14840     SLTYQALCLG --KGNYTVNL HFAEIMFNEK NM---YSNLG RRYFDIYVQG
AT1G53420     SLTYYALCLE --NGNYNVNL HFAEIMFNGN NN---YQSLG RRFFDIYIQR
AT2G22610     ---FCYQLNN LLPGEYLIDF HFAEIINTNG PK-------G IRVFNVYVQ-
AT1G29730     SLAYYAFCFE --NGSYNVKL HFAEIQFSDV EP---YTKLA KRVFNIYIQG
AT1G72250     ---FAYKFQS LDPGDYFIDL HFAEIEFTKG PP-------G V---------
AT1G51860     EPWLLWWTLD ENTAQSYVYM HFAEVQNLTA NE-------- TREFNITYNG
AT3G46240     GLSLVDPTLP SAGVPVYLAM YFSEP---LE SS-------- LRSFNIFFGG
AT2G23200     MNVTWSFKVK -SNHRHFIRI HFSDILSNLS NS-------- DSDFYLFVNG
AT2G14440     EPLTFTWMSE SSDDETYAYL YFAEIQQLKA NE-------- TRQFKILVN-
AT1G30570     NIS-WKFEVD -PNFDYLVRL HFCELLV--- ------DKQN QRIFRIYINN
AT3G05990     PLEFTWPPMP LKMATYYIAL YFAHDSDSMG DG-------- SRVFDVSVNG
AT3G46420     APLAIPGVLD FPSDKLYLFL HFSEIQVLKA NE-------- TREFEIFWNK
AT1G25570     -S-LVYELEV DAKLDYLLWF HFSEIDSTVK KA-------G QRVFDLVVN-
AT3G46370     ARLSFTENLE FPHDELYLYF HFSEVQVLQA NQ-------- SREFSILWNG
AT3G46270     GLIISDLPLP STATLVYLAL YFSEPQSLGR TQ-------- KRSFNVFLDD
AT4G39110     NVS-WNFPSN -PSFNYLIRL HFCDIVS--- ------KSLN DLYFNVYING
AT1G07560     SPLTISWNLE TPDDLVYAYL HVAEIQSLRE ND-------- TREFNISAGQ
AT1G51820     DTLNITWTVE PPTTQFYSYV HIAEIQALRA NE-------- TREFNVTLNG
AT1G24485     DMAFYDIKLP FSGVTFYIVI YFSEPLSLGS DQ-------- KRSFNVYYED
AT5G39030     NLT-WLFTVD -AGFSYLVRL HFFEKY---- -----LNKAN QRVFSIFLGN
AT3G46290     NVT-WEFDVD -PGFQYYFRF HFCDIVS--- ------LSLN QLYFNLYVDS
AT5G61350     NLS-WRMSVD -FGHDYFIRL HFCDIVS--- ------KSLN DLIFNVFINK
AT1G51805     ETLNITWNVE PPTTKVYSYM HFAELETLRA ND-------- TREFNVMLNG
AT5G59660     APLTIEWPSG SPS------- -----QEVPG TN-------- ITFF------
AT5G39000     NLT-WLFTVD -AGFSYLVRL HFCETLSE-- -----VNKEG QRVFSIFIEN
AT3G04690     NLT-WMFQID -KNFTYILRL HFCEFQ---- -----LSKIN QKVFNIYINN
AT1G51790     GFVDFGWIPD DPSLEFFFYL YFTELQQPNS GT------VE TREFVILLN-
AT5G28680     NLT-WMFQVD -TNFTYIMRL HFCEFQ---- -----LAKIN QKVFNIFINN
AT2G19190     LYLTLSFRPP DPNAKFYVYM HFAEIEVLKS --------NQ TREFSIWLNE
AT2G04300     APFNFTWSLI PSTAKFYSYM HFADIQTLQA NE-------- TREFDMMLNG
AT5G59680     APLTNEWSSV NPDEQYYVYA HFSEIQELQA NE-------- TREFNMLLNG
AT2G19210     QDIIFSWEPK DPTWKYFVYM HFAEVVELPS --------NE TREFKVLLNE
AT1G51890     QPLKINWSLD DITSQSYIYM HFAEIENLEA NE-------- TREFNITYNG
AT1G51880     QPLIFDWTLD NITSQSYVYM HFAEIQTLKD ND-------- IREFNITYNG
AT5G59616     ----MEWSSS NVNNQYYLYG HFAEIQELQT ND-------- TREFNMFWNR
AT2G28970     APLIITWKPR PSNAEVYFYL HFAEIQTLAA NE-------- TREFDIVFKG
AT5G54380     NVT-WVLPVD -PDFRYFVRV HFCDIVS--- ------QALN TLVFNLYVND
AT5G48740     -SLSYTLSLH TPG-DYYIIL YFAGILSLSP ---------- --SFSVTIND
AT2G28960     LPLRFYVSLD DDSDKVNVYF HFAEIQALRG NE-------- TREFDIELEE
AT3G21340     GTWNFSWLLP SSTTQFYVYM HFAEIQTLRS LD-------- TREFKVTLNG
AT3G46280     GLVLSDQTLP LTGVPVYLVL YFSEPQSLGR TQ-------- RRSFNVFLDN
AT3G46260     GFIMSGLNLP STLLPVYLAL YFSEPQSLGR TQ-------- KRSFTVFLDG
AT1G05700     RPITMTLENS DPNVRYFVYM HFAEVEDLSL KP------NQ TREFDISIN-
AT3G46400     VPLSFTKDLE FPKDKLYFYF HFSEIQPLQA NQ-------- SREFSILWNG
AT5G38990     NLT-WLFEVD -AGFAYIVRL HFCETQPE-- -----VNKTG DRVFSIFFGY
AT1G51800     AAMNISLVGV ERTVQFYVFM HFAEIQELKS ND-------- TREFNIMYNN
AT5G24010     NIS-WGFQVD EKRVLHLVRL HFCDIVS--- ------SSLN QLYFNVFINE
AT2G28990     APLIITWDPL PINAEVYLYM HFAEIQTLEA NE-------- TRQFDVILRG
AT4G20450     DTWSTTWSIQ NPDDDVHVYL HFAEIQALKP SD-------- TREFSILWNK
AT1G49100     GTWEFPWSLE DSTTRFHIYL HFAELQTLLA NE-------- TREFNVLLNG
AT4G29450     DPLELFWTSD DPNVRFYAYL YFAELETLEK --------NE TRKIKILWN-
AT1G51840     ---------- ---------- ---------- ---------- ----------
AT5G59670     APLTFKWNSE KLDVQYYFYA HYAEIQDLQA ND-------- TREFNILLNG
AT1G51850     DTLNITWTVE PPTTKFYSYM HFAELQTLRA ND-------- AREFNVTMNG
AT5G59700     NVT-WDFDVD -PGFQYFLRF HFCDIVS--- ------KALN QLYFNLYVDS
AT2G19230     EQIMVYWEPR DPNWKFYIYI HFAEVEKLPS --------NE TREFSVFLNK
AT1G51870     APWSLVWTID NTTALSYVYM HFAEIQDLKA ND-------- LREFDITYNG
AT3G46330     AALTMVWRLE NPDDQIYLYM HFSEIQVLKA ND-------- TREFDIILNG
AT2G29000     EPITVEYGGY SSGDQVYLYL HFAEIQTLKA SD-------- NREFDIVWAN
AT3G46350     PSYIGITFST SSEERFFIYL HFSEVQALRA NE-------- TREFNISING
AT2G37050     --LTYRMNLD GFPGFGWAFT YFAEIEDLAE DE-------- SRKFRLVLPE
AT1G07550     DPMTITWNLK TATDQVYGYI YIAEIMEVQA NE-------- TREFEVVVNN
AT1G51810     TTMEFPWNLE PPTSQFYLFL HFAELQSLQA NE-------- TREFNVVLNG
AT4G29990     NPLKFNWAPD DPRSKFYIYM HFAEVRELQR --------NE TREFDIYIN-
AT5G59650     APLTISWPPD NPGDQYYLYS HFSEIQDLQT ND-------- TREFDILWDG
AT1G28340     -PDLTYSLDV EPNRNYSVWL HFAEIDNTIT AE-------G KRVFDVVING
AT3G51550     NLT-WIFSID -SGFTYLVRL HFCEVSSN-- -----ITKIN QRVFTIYLNN
```

```
AT2G21480    NVT-WNFPSN -PSFHYFIRL HFCDIIS--- ------KSLN DLYFNVYING
AT5G16900    TRLTIIWTLD NPDEQIHLYV HFAELEPVGE NTDEALRTLF TRTFYFVVNG
AT1G67720    --ISYRLNLE DFPANARAYA YFAEIEELGA NE-------- TRKFKLVQPY
AT3G19230    PLELQWPPFP LPATKYYVAL YFQDNRSPGP MS-------- WRAFDVSVNG
AT1G51830    ATLNITWTIE PPTTPFYSYI HFAELQSLRA ND-------- TREFNVTLNG
AT5G39020    NLTGMFLTVD -AGYNYLVRL HFCETLPQ-- -----VTKAG QRVFSIFVED
AT4G00300    DMS-WGFKVN -VGYKYLIRM HFCDIAS--- ------KSLG RLYFNVYING
AT4G29180    EPLELSWTSS DPDTRFYAYL YFAELENLKR --------NE SREIKIFWN-
AT1G51910    HPLNIWWDLQ NINAPSYVYM HFAEIQNLKA ND-------- IREFNITYNG
AT3G46340    APFRFTEELD SPTDELYVYL HFSEVQSLQA NE-------- SREFDILWSG
AT2G14510    EPLTFTWNLE SSDDETYAYL FFAEIQQLKV NE-------- TREFKILAN-

                    360        370        380        390        400
             ....|....| ....|....| ....|....| ....|....| ....|....|
X.laevis     ---------- ---EEEEEEG STSKKQINKN RVQS---GPR TPNPYASDNS
CORK1        KLVEKDFD-- -MQKAANGSS IRVIQRVYKA NVSE---NYL EVHLFWAGKG
AT1G29750    KLVAKDFN-- -IMDEAKGAQ TPIIKPLT-A YVTN---HFL TIRLSWAGKG
AT1G53430    NLLERDFN-- -IAERAGGVG KPFIRQIDGV QVNG---STL EIHLQWTGKG
AT1G56120    RLVEKDFD-- -IRRTAGGSS VRAVQREYKT NVSE---NHL EVHLFWAGKG
AT1G07650    QLVIKNFN-- -IQEAARGSG KPIIKSFL-V NVTD---HTL KIGLRWAGKG
AT1G29720    KLFLRDFN-- -INKEANGNM KPVIKEIN-A TVTN---HML EIRLYWAGKG
AT1G56140    RLVEKDFD-- -VRRTAGDST VRAVQREYKA NVSQ---NHL EIHLFWAGKG
AT1G56130    RLVEKDFD-- -VRRTAGDST VRAVQRVYKA NVSE---NHL EVHLFWAGKG
AT1G53440    ILLERDFN-- -IAQRAGGVG KPFLRQVDEV QVNG---STL EIHLKWTGKG
AT1G29740    KLIWEDFS-- -IREEANGTH KEVIKEVN-T TVTD---NTL EIRLYWAGKG
AT3G14840    KREVKDFN-- -IVDEAKGVG KAVVKKFP-V MVTN---GKL EIRLQWAGKG
AT1G53420    KLEVKDFN-- -IAKEAKDVG NVVIKTFP-V EIKD---GKL EIRLYWAGRG
AT2G22610    DE-------- ---KATEFDI FSVVGANRPL LLVD-LRVMV MDDGLIRVRF
AT1G29730    KLIWEDFS-- -IREEANGTH KEVIREVN-T TVTD---NTL EIRLYWAGKG
AT1G72250    ---------- ----ISGLDL FSQVGANTPL VIED-LRMLV GREGELSIRL
AT1G51860    G--------- ---LRWFSYL RPPNLSISTI FNPR--AVSS -SNGIFNFTF
AT3G46240    K--------- ----QVGRGP VVPLFGKATQ VVVR----DV VASSSTLLTL
AT2G23200    YWR------- ---VDVKPSE QPRLASPFFK DVVN----VS DGSGLLNISI
AT2G14440    ---------- ---GVYYIDY IPRKFEAETL ITPA--ALKC GGGV-CRVQL
AT1G30570    QTAAGNFD-- IFAHAGGKNK GIYQDYLDPV SSK------N DVLWIQLGPD
AT3G05990    I--------- ----TYYKEL SVTPAGAVIF ASRW----PL EG--LTTLAL
AT3G46420    K--------- ----LVYNAY SPVYLQTKTI RNPS--PVTC ERGE-CILEM
AT1G25570    DN-------- ---NVSRVDV FHEVGG-FAA YSLN-YTVKN LSSTIVTVKL
AT3G46370    M--------- ----VIYPDF IPDYLGAATV YNPS--PSLC EVGK-CLLEL
AT3G46270    M--------- ----QVGSHP IVPVFGKATQ LVLR----DV EATSGSQIVL
AT4G39110    KTAISGLD-- LST-VAGNLA APYYKDIVVN ATL----MGP -ELQVQIGPM
AT1G07560    ---------- ---DVNYGPV SPDEFLVGTL FNTS--PVKC EGGT-CHLQL
AT1G51820    E--------- ----YTFGPF SPIPLKTASI VDLS--PGQC DGGR-CILQV
AT1G24485    K--------- ----QVGSDL IVPPFGAVTQ ASLR----DV VKTELAYLTF
AT5G39030    QMAREEM--- DVIRLSGGPR IPIYLDFRIY VGSE-SGPRP -DLRLDLHPL
AT3G46290    MVAATDID-- LSTLVDNTLA GAYSMDFVT- QTP----KGS NKVRVSIGPS
AT5G61350    LSAISALD-- LSS-LTSALG TAYYADFVLN AST----ITN GSILVQVGPT
AT1G51805    N--------- ----DLFGPY SPIPLKTETE TNLK--PEEC EDGA-CILQL
AT5G59660    ---------- ------SDPI IPKKLDITSV QSVT--PKTC QEGK-CSLQL
AT5G39000    QTATLEM--- DVFRMSGGSW IPMYLDYTVI AGSG-SGRRH -DLRLDLHPL
AT3G04690    RTAQADTTPA DIIGWTGEKG IPMYKDYAIY VDAN-NGG-- EEITLQMTPS
AT1G51790    -G-------- ---KSFGEPL SLNYFRTLAL FTSN-----P LKAESFQFSL
AT5G28680    RTAQGDTNPA DILGWTGGKG IPTYKDYAIY VDAN-TGGGG EEISLQMTPS
AT2G19190    DV-------- ---IS--PSF KLRYLLTDTF VTPD-----P VSGITINFSL
AT2G04300    N--------- ----LALERY RPKTFATGTI YFIK--PQIC EGGQ-CIIEL
AT5G59680    K--------- ----LFFGPV VPPKLAISTI LSVS--PNTC EGGE-CNLQL
AT2G19210    KE-------- ---IN-MSSF SPRYLYTDTL FVQN-----P VSGPKLEFRL
AT1G51890    G--------- ---ENWFSYF RPPKFRITTV YNPA--AVSS -LDGNFNFTF
AT1G51880    G--------- ---QNVYSYL RPEKFEISTL FDSK--PLSS -PDGSFSLSF
AT5G59616    Q--------- ----VIADPL IPPKFTIYTI FSQS--PSTC EGGK-CSFQL
AT2G28970    N--------- ----FNYSAF SPTKLELLTF FTSG--PVQC DSDG-CNLQL
AT5G54380    DLALGSLD-- LST-LTNGLK VPYFKDFISN GSV----ESS GVLTVSVGPD
AT5G48740    EVK------- ------QSDY TVTSSEAGTL YFTQ------ KGISKLNITL
AT2G28960    D--------- ----IIQSAY SPTMLQSDTK YNLS--PHKC -SSGLCYLKL
AT3G21340    K--------- ----LAYERY SPKTLATETI FYST--PQQC EDGT-CLLEL
AT3G46280    T--------- ----QVGSRP IVPVFGKATQ FILR----DV VATSASQIVF
AT3G46260    M--------- ----QVGSHP IVPVFGKATQ VVLR----DI MASSESQLVF
AT1G05700    -G-------- ---VTVAAGF SPKYLQTNTF FLN------P ESQSKIAFSL
AT3G46400    E--------- ----IIIPTL SPKYLKASTL YSVS--PFVC EVGK-CLLEL
AT5G38990    QLAMREM--- DVFRLSGGFR LPMYLDFKVL VDAD-GTSQR PSLRVDLTPY
AT1G51800    ---------- ---KHIYGPF RPLNFTTSSV FTPT--EVVA DANGQYIFSL
AT5G24010    YLAFKDVD-- LSTLTFHVLA SPLYIDFVAE S-----DRSG -MLRISVGPS
AT2G28990    N--------- ----FNHSGF SPTKLKVFTL YTEE--PMKC GSEG-CYLQL
AT4G20450    NT-------- ----IIRDYY SPLEFMADTV PIRT--SSKC GDDGFCSLDL
AT1G49100    K--------- ----VYYGPY SPKMLSIDTM SPQPDSTLTC KGGS-CLLQL
AT4G29450    GS-------- ---PVSETSF EPSSKYSTTF SNPR-----A FTGKDHWISI
AT1G51840    ---------- ---------- ---------- ---------- ----------
AT5G59670    QN-------- ---LSVTGPE VPDKLSIKTF QSSS--PISC NGWA-CNFQL
AT1G51850    I--------- ----YTYGPY SPKPLKTETI YDKI--PEQC DGGA-CLLQV
```

```
AT5G59700   MDVVENLD-- LSSYLSNTLS GAYAMDFVT- GSA----KLT KRIRVSIGRS
AT2G19230   EQ-------- ---IDTTSVF RPSYLYTDTL YVQN-----P VSGPFLEFVL
AT1G51870   G--------- ---KLWFSQF RPNKLSILTM FSQV--PLTS -SNGEYNFTF
AT3G46330   ET-------- ----INTRGV TPKYLEIMTW LTTN--PRQC NGGI-CRMQL
AT2G29000   N--------- ----IKKLAY KPKVSQIDTL LNTS--PNKC -DNTFCKAFL
AT3G46350   E--------- ----SVADLY RPVYL---VI YSPR--RKLS SKG------L
AT2G37050   QP-------- ---EYSKSVV NIKENTQRPY RVYAPGYPNI TLPFVLNFRF
AT1G07550   ---------- ---KVHFDPF RPTRFEAQVM FNNV--PLTC EGGF-CRLQL
AT1G51810   N--------- ----VTFKSY SPKFLEMQTV YSTA--PKQC DGGK-CLLQL
AT4G29990   -D-------- ---VILAENF RPFYLFTDTR STVD-----P VGRKMNEIVL
AT5G59650   A--------- ----VVEEGF IPPKLGVTTI HNLS--PVTC KGEN-CIYQL
AT1G28340   DT-------- ---FFEDVDI IKMSGGRYAA LVLN-ATVTV SGRTLTVVLQ
AT3G51550   QTAEPEA--- DVIAWTSSNG VPFHKDYVVN PPEG-NGQQD --LWLALHPN
AT2G21480   KTAISGLD-- LST-VAGDLS APYYKDIVVN STL----MTS -ELQVQIGPM
AT5G16900   K--------- ---ISYDESI TPLDLAVSTV ETVV--N-KC DGGN-CSLQL
AT1G67720   FP-------- ---DYSNAVV NIAENANGSY TLYEPSYMNV TLDFVLTFSF
AT3G19230   L--------- ----SFLRKL NVSTNGVMVY SGQW----PL SG--QTQITL
AT1G51830   E--------- ----YTIGPY SPKPLKTETI QDLS--PEQC NGGA-CILQL
AT5G39020   KMAKKET--- DVIRLSGGPR IPMYLDFSVY VGFE-SGMIQ PELRLDLVPL
AT4G00300   NLAYEDFD-- ISYAADNVLA SPYYIDFVVD ATADDNNPSG SSITVSVGPS
AT4G29180   GS-------- ---PVSG-AF NPSPEYSMTV SNSR-----A FTGKDHWISV
AT1G51910   G--------- ---QVWESSI RPHNLSITTI SSPT--ALNS -SDGFFNFTF
AT3G46340   E--------- ----VAYEAF IPEYLNITTI QTNT--PVTC PGGK-CNLEL
AT2G14510   ---------- ---GVDYIDY TPWKFEARTL SNPA--PLKC EGGV-CRVQL

                    410        420
            ....|....| ....|....| ...
X.laevis    SLMFPILVAF GVFIPTLFCL CRL
CORK1       TCCIPAQGTY GPLVSAI--- ---
AT1G29750   TTRIPTRGVY GPIISAI--- ---
AT1G53430   TNVIPTRGVY GPLISAI--- ---
AT1G56120   TCCIPIQGAY GPLIAAV--- ---
AT1G07650   TTGIPIRGVY GPMISAI--- ---
AT1G29720   TTLIPKRGNY GPLISAI--- ---
AT1G56140   TCCIPIQGAY GPLISAV--- ---
AT1G56130   TCCIPIQGAY GPLISAV--- ---
AT1G53440   TNVIPTRGVY GPLISAI--- ---
AT1G29740   TTIIPKRGNY GSLISAI--- ---
AT3G14840   TQAIPVRGVY GPLISAV--- ---
AT1G53420   TTVIPKERVY GPLISAI--- ---
AT2G22610   EGINGS---- -PVVCGI--- ---
AT1G29730   TMIIPQRGYY GSLISAV--- ---
AT1G72250   EGVTGA---- -AILCGI--- ---
AT1G51860   AMTGNS--TL PPLLNALEIY TV-
AT3G46240   WSTSSA--LL PPMINAAELY VI-
AT2G23200   GTKEAN--KD AGFLNGLEMM EV-
AT2G14440   SKTPKS--TL PPQMNAIEIF SV-
AT1G30570   SSVGASG--- DALLSGLEIF KL-
AT3G05990   SPRSGS--NL PPLINGGEMF EL-
AT3G46420   IKTERS--TL PPLLNAVEVF TV-
AT1G25570   SSVSGA---- -PIISGLENY AI-
AT3G46370   ERTQKS--TL PPLLNAIEVF TV-
AT3G46270   KSTDDS--VL PTMINGLELY SI-
AT4G39110   G-EDTGT--K NAILNGVEVL KM-
AT1G07560   IKTPKS--TL PPLLNAIEAF IT-
AT1G51820   VKTLKS--TL PPLLNAIEAF TV-
AT1G24485   EATPDS--TL DPLINALELY VI-
AT5G39030   VKDNPEY--Y EAILNGVEIL KL-
AT3G46290   T-VHTDY--P NAIVNGLEIM KM-
AT5G61350   PNLQSGK--P NAILNGLEIM KL-
AT1G51805   VKTSKS--TL PPLLNAIEAF TV-
AT5G59660   TRTNRS--TL PPLLNALEIY AV-
AT5G39000   VSINPKY--Y DAILNGVEIL KM-
AT3G04690   TFGQPEY--Y DSSLNGLEIF KM-
AT1G51790   RQTQ-S-SSL PPLINAMETY FV-
AT5G28680   TFGQPEY--Y DSQLNGLEIF KI-
AT2G19190   LQPPGE-FVL PPIINALEVY QV-
AT2G04300   LKTSKS--TL PPLCSALEVF TV-
AT5G59680   IRTNRS--TL PPLLNAYEVY KV-
AT2G19210   QQTP-R-STL PPIINAIETY RV-
AT1G51890   SMTGNS--TH PPLINGLEIY QV-
AT1G51880   TKTGNS--TL PPLINGLEIY KV-
AT5G59616   RRTNRS--TL PPLLNAFEVY TV-
AT2G28970   VRTPNS--TL PPLINALEAY TI-
AT5G54380   S--QADI--T NATMNGLEVL KI-
AT5G48740   RKIKFN---- -PQVSALEVY EI-
AT2G28960   VRTPRS--TL PPLISAIEAF KV-
AT3G21340   TKTPKS--TL PPLMNALEVF TV-
AT3G46280   QSTDDS--VL PPLINGLELY SI-
```

```
AT3G46260  KSTDDS--GL PTIISGLEVY SI-
AT1G05700  VRTP-K-STL PPIVNALEIY VA-
AT3G46400  KRTQNS--TL PPLLTAIEVF TV-
AT5G38990  KEDYPTY--Y DAILSGVEIL KL-
AT1G51800  QRTGNS--TL PPLLNAMEIY SV-
AT5G24010  DLSNPAR--V NALLNGVEIM RI-
AT2G28990  VKTPNS--TL PPLINAIEAY SV-
AT4G20450  TRTKSS--TL PPYCNAMEVF GL-
AT1G49100  VKTTKS--TL PPLINAIELF TV-
AT4G29450  QKTV-D-STL PPILNAIEIF TA-
AT1G51840  ---------- ---------- ---
AT5G59670  IRTKRS--TL PPLLNALEVY TV-
AT1G51850  VKTLKS--TL PPLLNAIEAF TV-
AT5G59700  S-VHTDY--P TAILNGLEIM KM-
AT2G19230  RQGV-K-STR PPIMNAIETY RT-
AT1G51870  EMTSNS--TL PPLINALEIY TG-
AT3G46330  TKTQKS--TL PPLLNAFEVY SV-
AT2G29000  VRTQRS--TL PPLLNAYEVY IL-
AT3G46350  TGTIAA--DI QY-------- ---
AT2G37050  AKTADS--SR GPILNAMEIS KY-
AT1G07550  IKTPKS--TL PPLMNAFEIF TG-
AT1G51810  VKTSRS--TL PPLINAMEAY TV-
AT4G29990  QRTG-V-STL PPIINAIEIY QI-
AT5G59650  IKTSRS--TL PSLLNALEIY TV-
AT1G28340  PKAGGH---- -AIINAIEVF EI-
AT3G51550  PVNKPEY--Y DSLLNGVEIF KM-
AT2G21480  G-EDTGK--K NAILNGVEVL KM-
AT5G16900  VRSEASPGVR VPLVNAMEAF TA-
AT1G67720  GKTKDS--TQ GPLLNAIEIS KY-
AT3G19230  TPAKDA--PV GPFINAGEVF QI-
AT1G51830  VETLKS--TL PPLLNAIEAF TV-
AT5G39020  KDTNQTY--Y DAILSGVEIL KL-
AT4G00300  NKTSVDGNGV DAILNGVEIM KM-
AT4G29180  QKTA-E-STR PPILNAIEIF SA-
AT1G51910  TMTTTS--TL PPLINALEVY TL-
AT3G46340  KRTKNS--TH PPLINAIEFY TV-
AT2G14510  SKTPKS--TL PPLMNAIEIF SV-
```

Supplementary Figure S2. Alignment of malectin domains (MD) and malectin-like domains (MLD) in *A. thaliana,* and the malectin in *X. laevis*. Black shade indicates conserved amino acid residues over 90% threshold.

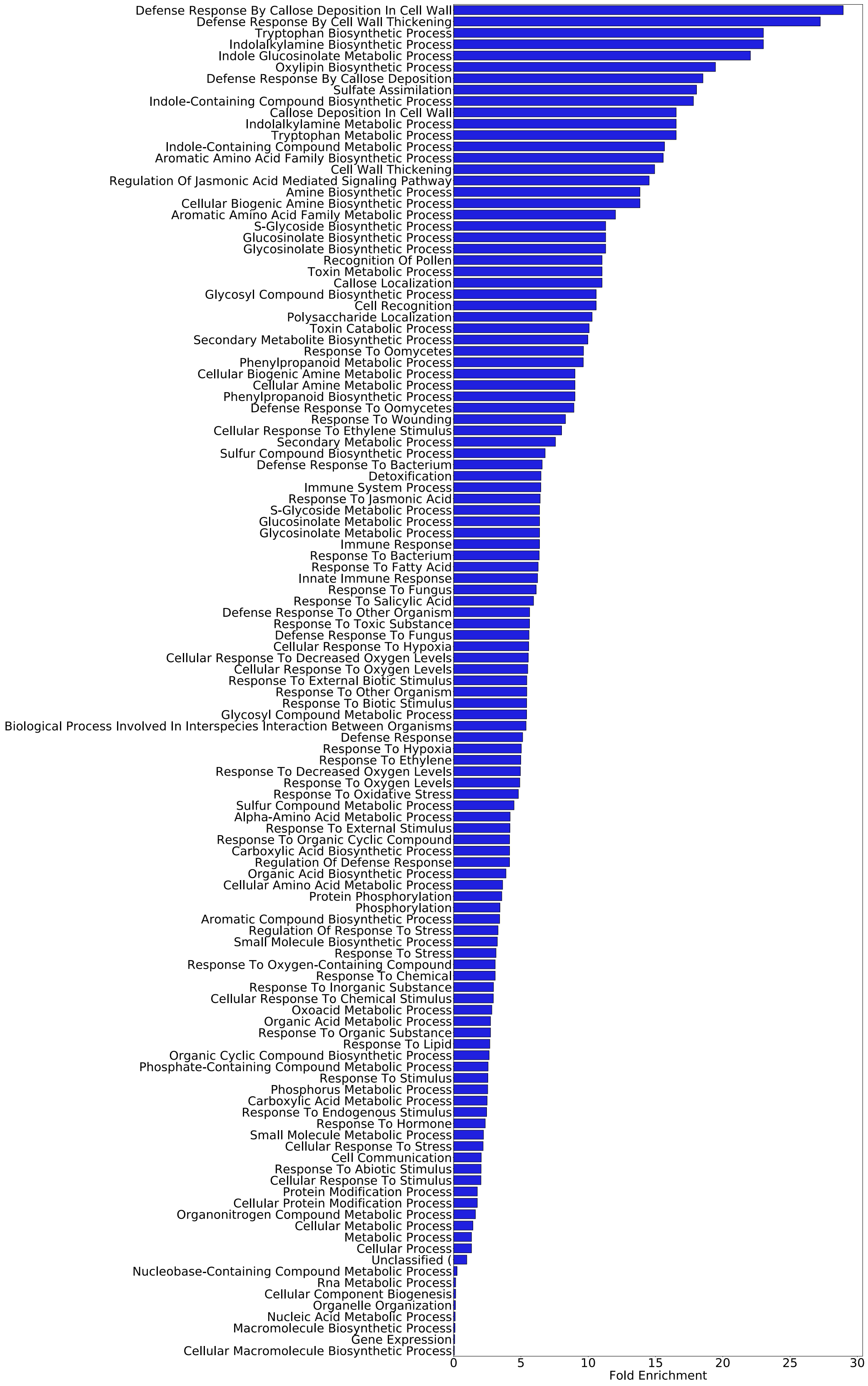

Supplementary Figure S3. Biological functions enriched from up-regulated genes by 10 µM CT compared to water control in root tissue of *cork1-2* segregated wild-type from the cross to aequorin wild-type.

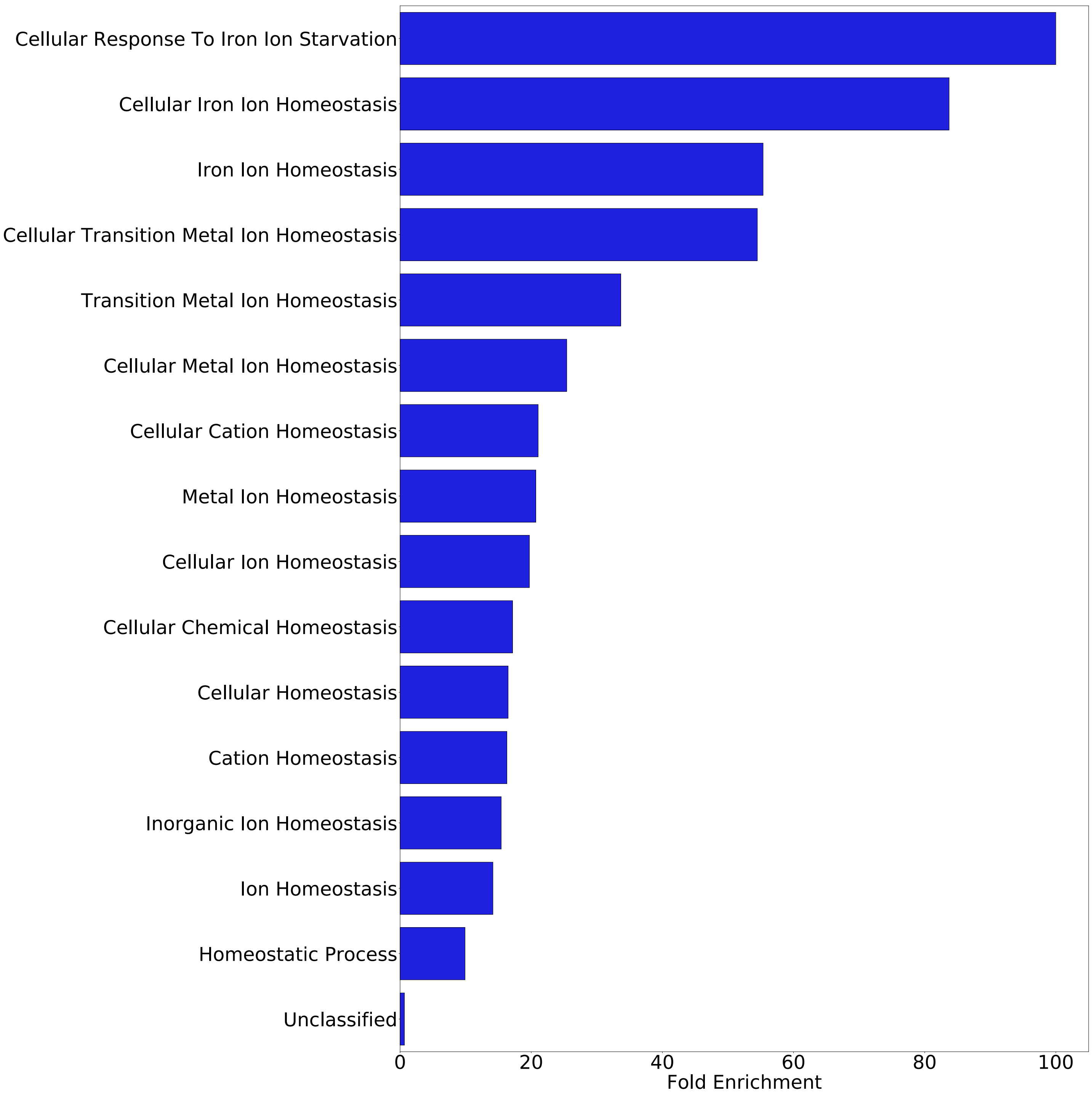

Supplementary Figure S4. Biological functions enriched from down-regulated genes by 10 µM CT compared to water control in root tissue of *cork1-2* segregated wild-type from the cross to aequorin wild-type.

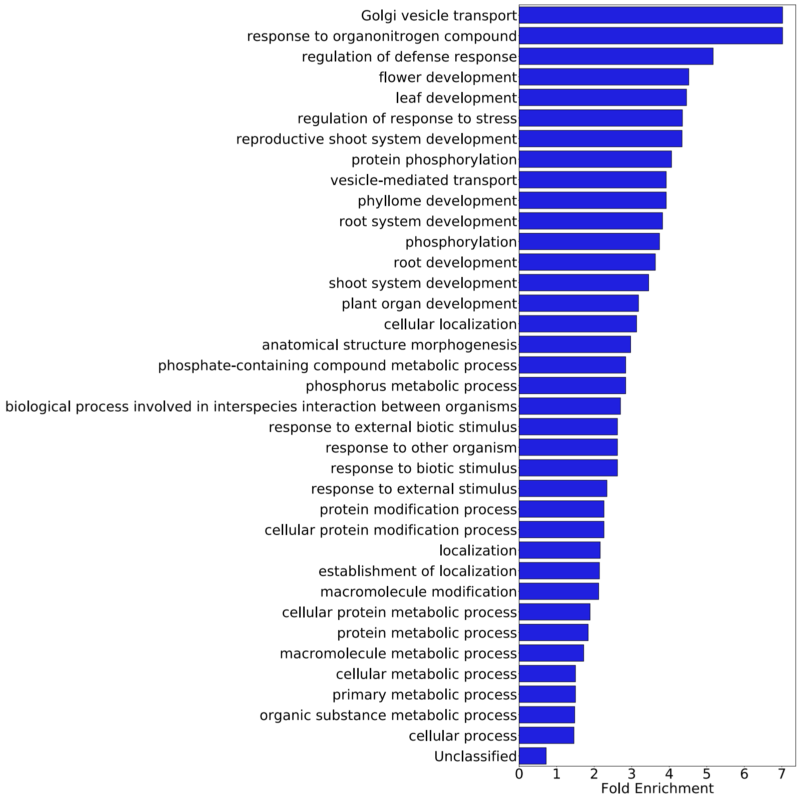

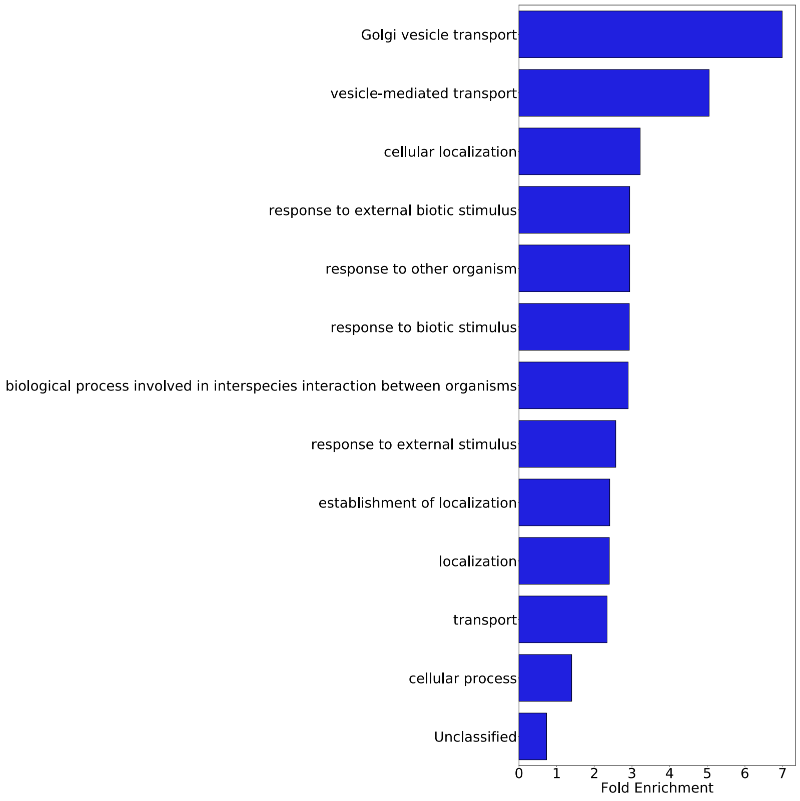

Supplementary Figure S5. Biological functions enriched from phosphoproteomic analysis between 10 µM CT compared to water control in root tissue of *cork1-2* segregated wild-type from the cross to aequorin wild-type. Top: 5 minutes after treatment; Bottom: 15 minutes after treatment.

Supplementary Table S1. Primers used in this study (5'→3').

Primers for genotyping:		
cork1-1 LP	TCTATTTAACCCGGTTCCACC	
cork1-1 RP	GAATCGACAAAAGAGCAGTCG	
cork1-2 LP	CTGCTGTATTCCTGCTCAAGG	
cork1-2 RP	TGATATAGTGGCAATCTCCGC	
LB_SALK	GACCGCTTGCTGCAACTCTCTCAGG	
Primers for gene expression analysis by quantitative PCR (qPCR):		
RPS (AT1G34030)	RPS-qF	GTCTCCAATGCCCTTGACAT
	RPS-qR	TCTTTCCTCTGCGACCAGTT
PER4 (AT1G14540)	PER4-qF	ATGTGAAGGTTGGTCGAAGAG
	PER4-qR	ATGTGTGAGCTCCTGAGAGAG
MYB51 (AT1G18570)	MYB51-qF	CTACAAGTGTTTCCGTTGACTCTGAA
	MYB51-qR	ACGAAATTATCGCAGTACATTAGAGGA
CORK1 (AT1G56145)	CORK1-qF	ACCAGAGTACGTGATGCTTG
	CORK1-qR	TCCATGCCCATTCGAGAAG
CCOAOMT (AT1G67980)	CCOAOMT-qF	TGGTGAACGACAAATGTGAGTTTG
	CCOAOMT-qR	ACAAAACCAAACCACAAGGTGTTG
WRKY40 (AT1G80840)	WRKY40-qF	AGCCCTCCCAAGAAACGCAAATC
	WRKY40-qR	GCTTGGAGCACAAGCACATTTGAAG
PR-1-Like (AT2G19990)	AT2G19990-qF	ACGATTATGATAGTAACACGTGTG
	AT2G19990-qR	AGGATCATAGCTACAAATCACC
PEN2 (AT2G44490)	PEN2-qF	AAGCCATCCATGAAGATGGAG
	PEN2-qR	ACCATATCCACTGTTCCACTC
CYP71B15/PAD3 (AT3G26830)	PAD3-qF	ACGAGCATCTTAAGCCTGGA
	PAD3-qR	TCGGTCATTCCCCATAGTGT
TSA1 (AT3G54640)	TSA1-qF	TCAGTCGCTCTTGAAGGATATC
	TSA1-qR	TCCAGCTATCTGTTTCACATGC
PMR4 (AT4G03550)	PMR4-qF	CTGGAATGCTGTTGTCTCTGTTG
	PMR4-qR	TCGCCTTTTGATTTCTTCCCAGT
WRKY30 (AT5G24110)	WRKY30-qF	CGGAGCCAAATTTCCAAGAGG
	WRKY30-qR	GACGGAGAGTTTGATGCTGAG
FLS2 (AT5G46330)	FLS2-qF	CGCAGAACAATCTCTCGGGT
	FLS2-qR	CGTCATGTTCCCGAAGCTCT
CYP81F2 (AT5G57220)	CYP81F2-qF	GTGAAAGCACTAGGCGAAGC
	CYP81F2-qR	ATCCGTTCCAGCTAGCATCA
Primers for plasmid construction:		
35S::CORK1 in pB7FWG2.0	CORK1-caccF	CACCATGCTGAGATTAATTCTCTCCTTG
	CORK1-R	TCATCAATGTCGTCGTCCATGTTCTTC
	CORK1-NoSTOP	ATGTCGTCGTCCATGTTCTTC
35S::ARF1 in pB7FWG2.0	ARF1-caccF	CACCATGGCAGCTTCCAATCATTCATC
	ARF1-R	TCATCATCTTGATCCCGCCATAGATG
CORK1^{KD}	BamHI-KD-F	AAAAAAGGATCCAGGAAAAGAAAAAGGGCGGCT
	EcoRI-KD-R	AAAAAAGAATTCTCATCAATGTCGTCGTCCATGTTCTTCAAC
Primers for site-directed mutagenesis:		
G748E-F	TTGTATGAATGCTGCATTGAGG	
G748E-R	TGCAGCATTCATACAATTTTACAAGG	
F520A-F	TGTAACGGTTCAGGCGGCTGAGATACAAATAC	
F520A-R	GTATTTGTATCTCAGCCGCCTGAACCGTTACA	
F539A-F	CTTGGAAGGCGAATTGCGGACATATATGTCCAG	
F539A-R	CTGGACATATATGTCCGCAATTCGCCTTCCAAG	

Supplementary Table S2. UniProt accession number of amino acid sequences used in the multiple alignment for the *Arabidopsis* MD/MLD domains.

F4HSE1	Q9ASQ6	F4I336	F4I337	Q9FXF2	C0LGG7	F4HRH4	C0LGG9	F4I3K0
C0LGH2	C0LGH3	F4I3K4	F4IBQ9	F4IJK6	C0LGN2	C0LGD6	C0LGD8	C0LGD9
F4I9A5	F4ICJ5	F4HWL3	Q9SA72	F4I065	F4IB60	Q9C8I6	F4IB63	Q9FZB8
C0LGG3	Q9FZB6	F4IB68	F4IB69	C0LGG4	F4IB71	Q9FZB1	C0LGG6	F4IB76
C0LGI2	Q9SI06	Q9ZQQ7	Q9ZQR3	O64483	O65924	O64556	Q9SJT0	O22187
C0LGL4	O81067	O81069	F4IJP7	F4IPZ3	O80623	Q9SR05	Q9SFG3	F4JB46
Q9LIG2	F4J800	F4J801	Q67ZF1	Q8GYH9	Q9LX66	C0LGP2	Q9SNA3	F4J810
Q9SNA0	F4J927	Q9SN97	Q9SCZ4	Q0WQL0	C0LGQ7	F4JMW3	Q9M0D8	Q9SZV2
Q9T020	C0LGT5	Q9FLW0	Q3E8W4	Q9FID9	Q9FID8	Q9FID6	Q9FID5	C0LGV0
Q9LK35	F4KJ89	C0LGW2	F4KJ91	Q9FN94	Q9FN93	Q9FN92	Q9FLJ8	Q6INX3
